# Benchmarking large language models for HIV medical decision support

**DOI:** 10.1038/s43856-026-01875-1

**Published:** 2026-08-29

**Authors:** Gonzalo Cardenal-Antolin, Jacques Fellay, Bashkim Jaha, Huldrych F. Günthard, Johannes Nemeth, Roger Kouyos, Niko Beerenwinkel, Diane Duroux

**Affiliations:** 1 Department of Biosystems Science and Engineering, ETH Zurich, Basel, Switzerland; 2 School of Life Sciences, École Polytechnique Fédérale de Lausanne, Lausanne, Switzerland; 3 SIB Swiss Institute of Bioinformatics, Lausanne, Switzerland; 4 Biomedical Data Science Center, Lausanne University Hospital and University of Lausanne, Lausanne, Switzerland; 5 Department of Infectious Diseases and Hospital Epidemiology, University Hospital Zurich, Zurich, Switzerland; 6 Institute of Medical Virology, University of Zurich, Zurich, Switzerland; 7 ETH AI Center, ETH Zurich, Zurich, Switzerland; 8 Department of Quantitative Biomedicine, University of Zurich, Zurich, Switzerland

**Keywords:** Computational biology and bioinformatics, HIV infections

## Abstract

**Background:**

Large language models (LLMs) are emerging as tools to support clinical decision making. HIV management is a compelling use case due to its complexity and dynamic nature, involving diverse treatment options, comorbidities, and adherence challenges. However, integrating LLMs into clinical practice raises concerns about accuracy, safety, and clinician acceptance. Despite growing interest, their performance in HIV care remains poorly studied, and benchmarking is lacking.

**Methods:**

We developed HIVMedQA, a clinician-curated benchmark of HIV-related open-ended medical question-answer pairs spanning basic knowledge, clinical reasoning, complex patient vignettes, and bias-modified scenarios. We evaluated seven general-purpose and three medical LLMs. Performance was assessed using lexical similarity and an extended medical LLM-as-a-judge framework capturing key clinical dimensions, including question comprehension, reasoning, knowledge recall, bias, potential harm, and factual accuracy, to better capture nuances relevant to the medical domain, with additional evaluation by HIV-experienced physicians.

**Results:**

Performance varies substantially across models and task complexity. Gemini 2.5 Pro achieves the highest overall scores, followed by Claude 3.5 Sonnet and MedGemma-27B. Knowledge recall is generally stronger than question comprehension or clinical reasoning. Medical LLMs do not consistently outperform general-purpose models, and model size alone does not predict performance. Several models are sensitive to cognitive bias prompts. LLM-as-a-judge scoring aligns better with clinician assessment than lexical metrics.

**Conclusions:**

HIVMedQA provides a structured benchmark for evaluating LLMs in HIV clinical decision support. Current LLMs show promise, but limitations in reasoning, bias robustness, and safety indicate that careful validation, domain-specific evaluation, and clinician oversight remain essential before clinical deployment.

## Plain language summary

Large language models are increasingly being explored to support clinical decision-making, particularly where specialist access is limited. HIV care is complex, requiring integration of evolving therapies, diagnostic data, and patient history. We evaluated multiple AI models using a set of realistic HIV-related questions, assessing accuracy, safety, and clinical usefulness against expert judgment. While several models performed well on simpler tasks, only a few remained reliable for more complex scenarios, with many showing weaknesses in reasoning and susceptibility to misleading information. These results suggest that, although promising, current AI tools are not yet ready for routine clinical use and require further refinement and rigorous evaluation for safe deployment.

## Abstract (web summary)

Cardenal-Antolin et al., develop HIVMedQA, a clinician-curated benchmark of HIV-related open-ended question-answer pairs, and use it to evaluate general-purpose and medical large language models across knowledge, reasoning, bias, safety and accuracy dimensions. Gemini 2.5 Pro performs best overall, reasoning and bias robustness remain limited, and clinician-aligned LLM-as-a-judge assessment more accurately reflects model performance than lexical metrics alone.

## Introduction

Recent advances in large language models (LLMs) have opened the door to a broad range of applications in healthcare^1^. These include routine tasks such as generating discharge summaries from electronic medical records^2–4^, as well as more complex functions like medical question answering, clinical decision support, patient management planning, and diagnostic reasoning^5–7^. Notably, LLMs have surpassed the average human score on the United States Medical Licensing Examination (USMLE), with accuracy rising from 38.1% in 2021 to 95.8% in 2025^8^. This growing capability has spurred interest in leveraging LLMs to replicate curbside consults, a common practice in which a physician informally seeks clinical advice or clarification from a colleague regarding patient care or academic questions^9,10^. This paper focuses specifically on that use case. Medical LLM-based chatbots offer the potential to deliver rapid, expert-level input on demand, making them especially valuable to junior clinicians or practitioners managing unfamiliar conditions^11^. Their utility becomes even more pronounced in low-resource or remote settings, where access to specialists is limited. As global health systems face mounting strain^12,13^, and more than 40% of the world’s population experiences restricted access to care^14^, scalable tools that support real-time clinical decision-making are urgently needed.

Despite their promise, AI-driven generative chatbots come with significant risks^6^. These models are not designed to truly understand language. Rather, they operate by learning statistical associations between words. This limitation, combined with training data sourced from unverified and non-curated websites and books, raises serious concerns about the accuracy of their outputs. Furthermore, because responses are generated based on probabilistic patterns rather than genuine comprehension, issues with coherence and contextual relevance are common. These factors contribute to the problem of hallucinations, i.e., confident, fluent responses that are factually incorrect or misleading^15^. In the clinical context, such errors are referred to as confabulations^16^: statements that may range from plausible to nonsensical but are delivered with authoritative language, making them difficult to detect. Concerns also extend to generalizability, with studies highlighting limitations in performance across diverse patient populations^17,18^. Additionally, there are challenges related to the temporal relevance of information^6^. Since pretraining datasets are fixed in time, models risk becoming outdated in a field like medicine, which evolves rapidly. Ensuring alignment with current clinical guidelines and evidence requires continuous model updates and real-time knowledge integration.

Clinical practice is far more complex than simply answering examination questions correctly, making it difficult to identify benchmarks that accurately reflect the clinical utility of LLMs. Although recent results have been promising^19,20^, key questions remain. For example, while some emerging evaluation frameworks attempt to simulate clinical settings through doctor-patient conversations with AI agents^19,21,22^, the dominant approach still relies on multiple-choice medical exam-style questions^23–25^. However, this format fails to capture the real-world complexity of open questions, uncertainty, and nuance inherent in clinical decision making. As a result, these limitations contribute to ongoing mistrust and skepticism about the readiness of LLMs for clinical deployment^16^.

There is currently no consensus on the most appropriate way to evaluate AI-generated medical responses, as multiple types of performance metrics exist, including lexical matching, LLM-as-a-judge approaches, and neural evaluation techniques^26^. Each of these methods captures different aspects of performance, and their ability to account for the specific challenges of open-ended medical question answering remains debated. Importantly, evaluating medical question answering must go beyond factual accuracy alone. Dimensions such as clinical reasoning, potential for harm, and implicit bias must also be assessed to ensure that the outputs are not only correct but also trustworthy, safe, and equitable. Additionally, while it is often assumed that general-purpose models are less capable than domain-specific (e.g., medical LLMs) fine-tuned models, recent studies have challenged this notion, showing that general-purpose models can match or even outperform specialized counterparts in certain clinical tasks^27,28^.

It has been emphasized that rigorous evaluation and context-specific validation are essential to demonstrate the effectiveness and clinical utility of AI models in healthcare settings^6,25,29^. Human immunodeficiency viruses (HIV) care, in particular, presents a uniquely complex challenge. The chronic nature of the disease, the presence of opportunistic infections, comorbidities in immunocompromised patients, risk of drug resistance, and its disproportionate burden on marginalized populations all contribute to this complexity. Clinicians must integrate a wide array of clinical data, including genotypic resistance testing, viral load measurements, CD4 counts, prior treatment history, and comorbidity profiles, into their decision-making. They must also carefully balance a range of factors, including side effects, evolving treatment options, adherence issues, and the need for individualized care strategies^30–33^. These intricacies make HIV management a compelling domain for AI support, where large-scale data analysis and personalized intervention recommendations can enhance decision-making and improve outcomes.

Our findings provide a nuanced picture of the capabilities required for LLM-assisted curbside consultation in HIV care. Performance depends not only on factual knowledge but also on the ability to interpret clinical questions, reason across increasingly complex scenarios, and remain robust to potentially biasing contextual information. The observed variation across model types and sizes further suggests that neither medical specialization nor scale alone guarantees stronger clinical performance. Our results also demonstrate the value of clinically oriented evaluation frameworks that assess the quality of model responses beyond surface-level textual similarity. These findings identify priorities for improving and evaluating LLMs intended for clinical decision support.

## Methods

### General and medically fine-tuned large language models

As the landscape of LLMs continues to evolve, we aim to evaluate their usability in clinical contexts and explore their relevance in open-form medical questions beyond traditional closed-form medical question answering. To this end, we included ten well-established and state-of-the-art models covering large-scale and small-scale, proprietary and open-weight models (Table 1).

**Table 1:** Properties of the LLMs assessed, sorted by number of parameters

Model	Number of parameters	Open	Medical
Llama 3.2-Instruct	1B	Yes	No
Llama 3.1-Instruct	8B	Yes	No
MedGemma	27B	Yes	Yes
Gemma 3	27B	Yes	No
Llama 3.3-Instruct	70B	Yes	No
Meditron 3	70B	Yes	Yes
Med42 v2	70B	Yes	Yes
NVLM-D	72B	Yes	No
Gemini 2.5 Pro	Not disclosed	No	No
Claude 3.5 Sonnet v2	Not disclosed	No	No

We evaluated general-purpose models, including Gemma 3-27B, Gemini 2.5 Pro, Claude 3.5 Sonnet v2 (version 2024.10.22), Llama 3.3-70B-Instruct^34^, Llama 3.1-8B-Instruct, Llama 3.2-1B-Instruct, and NVLM-D-72B^35^. We also assessed the medical LLMs Meditron 3-70B^23^, MedGemma-27B (text-only model), and Med42 v2-70B^36^. GPT-4o was not included in this evaluation as it served as an evaluator. The default parameters were used (Supplementary Section 9).

For each model and each medical question, the same system prompt was leveraged. A system prompt acts as a guiding framework, shaping the behavior and style of the LLM throughout the interaction. It is designed to improve the model’s alignment with specific objectives, enhance the user experience, better uphold ethical guidelines, and maintain consistency in responses. Building on ref. ^23^, the following system prompt was applied (Supplementary Section 8):

“*You are a helpful, respectful and honest senior physician specializing in HIV. You are assisting a junior clinician answering medical questions. Keep your answers brief and clear. If a question does not make any sense, or is not factually coherent, explain why instead of answering something not correct. If you don’t know the answer to a question, please don’t share false information.*”

To assess whether RAG can offset smaller model size, we evaluated the effect of RAG on two models: Llama 3.1-8B-Instruct and MedGemma-27B. The retrieval corpus comprised major HIV clinical practice guidelines from U.S., WHO, and European organizations. Details on corpus preprocessing and retrieval are provided in Supplementary Section 7. For each query, we appended the following instruction to the prompt: *"Use the following clinical guideline excerpts to answer the question. If the excerpts are not sufficient, say so and answer using best medical judgment”*.

### HIV questionnaire

To assess the performance of LLMs in clinical HIV scenarios, our multidisciplinary team, comprising a general practitioner, an infectious disease clinician, AI researchers, and computational biologists, developed a questionnaire encompassing four categories, capturing increasing clinical complexity and potential cognitive biases. For each question, we provided an expert-validated gold-standard answer. Five iterations were performed for each model and each question. The categories are defined as follows:

Category 1 contains eleven questions assessing fundamental knowledge about HIV, such as *How is HIV diagnosed?*, or *How is HIV transmitted?*

Category 2 includes standard patient-level questions on clinical knowledge of HIV. The questions were selected from the USMLE^37,38^ Step 1 and adapted from multiple-choice questions into an open-question format. The questions were obtained by filtering for the terms *HIV* or *AIDS*. This process yielded 143 questions. From this set, ten questions that aligned with the open-question format were chosen and reformatted accordingly. For instance, *A 27-year-old man presents with a 2-week history of fever, malaise, and occasional diarrhea. On physical examination, the physician notes enlarged inguinal lymph nodes. An HIV-1 detection test is positive. Laboratory studies show a CD4+ count of* 650/*mm*^3^. *This patient is most likely currently in what stage of HIV infection?*

Category 3 consists of complex clinical vignettes that assess in-depth clinical knowledge and patient-level decision-making. The questions were obtained from USMLE Step 2 and 3. As in Category 2, the questions were filtered by *HIV* or *AIDS*, which yielded 139 questions. From these questions, 21 suitable for open-ended responses were selected and reformatted into an open-question structure. A vignette can include presenting symptoms, past medical history, social history, vital signs, physical examination findings, laboratory results, and imaging findings. Category 3 corresponds best to the level of complexity seen in clinics. An example of a question is provided in Supplementary Section 6.

Category 4 consists of Category 3 questions modified to introduce false information in the form of one of the three major clinical cognitive biases commonly observed in clinical decision-making^39^: recency bias, frequency bias, and status quo bias. Recency bias refers to the tendency to give more weight to recent events or information, often at the expense of older but possibly more relevant data. Frequency bias corresponds to the tendency to believe something is more common or likely simply because it was encountered more often. Status quo bias is the preference for the current state of affairs, leading people to resist change even when alternatives may offer benefits. An example of a question is provided in Supplementary Section 6.

### Evaluation

We used two types of open-QA performance metrics: LLM-as-a-judge and lexical matching.

#### LLM-as-a-judge: MedGPT

LLM-as-a-judge performance metrics have been extensively leveraged to evaluate LLM answers^40–43^. They refer to using an LLM as an automated evaluator of the output of other models. The LLM is prompted with a question, the AI-generated answer, and a gold-standard answer. It compares the AI-generated answer to the reference and assigns a score on a scale from 0 to 5, based on evaluation criteria defined in the prompt (see Supplementary Section 3). The advantages of this approach are its scalability and speed compared to human annotation. It also enables evaluation across multiple nuanced dimensions.

We used GPT-4o (gpt-4o-2024-08-06) to develop an evaluation metric, termed *MedGPT*, based on the prompt proposed by Wang et al.^44^ and informed by prior LLM-based evaluation frameworks^19,45^. We adapted the original framework, which focused on reading comprehension, reasoning, and knowledge recall, to additionally assess demographic bias and potential harm^46^. The five resulting scores, MedGPT1 to MedGPT5, correspond to distinct evaluation dimensions. MedGPT1 assesses question comprehension and the absence of misinterpretation; MedGPT2 evaluates the quality and clinical validity of the reasoning steps; MedGPT3 measures the accuracy and relevance of recalled medical knowledge; MedGPT4 assesses the presence of bias toward demographic groups; and MedGPT5 evaluates the potential severity and extent of harm associated with the response. Together, these dimensions provide a multidimensional assessment of response quality that extends beyond factual correctness alone. The prompt is available in Supplementary Section 3.

To improve MedGPT’s performance, we tested several prompt formulations and selected the version that produced the highest-scoring rephrased gold-standard answers. We iterated on the opening instruction, making it more evidence-based, discouraging guesswork, and anchoring it to explicit evaluation criteria. In parallel, we compared alternative scoring rubrics. For each rubric, we (1) defined the general purpose, (2) supplied descriptions, and (3) detailed how points should be deducted. For instance, instead of broadly flagging “incorrect reasoning,” the refined rubric subtracts points for specific issues such as logical fallacies, unclear rationale, or departures from accepted medical principles.

MedGPT evaluates model responses using the original question, the model-generated answer, and the gold-standard answer as context. To assess the impact of including the reference answer, we compared evaluation scores obtained with and without the gold-standard, thereby simulating a supervised versus unsupervised scoring setup. The prompt used for the unsupervised evaluation was identical to the one employed in the supervised setting, except that the reference answer was omitted. In other words, the model was provided only with the clinical question, the candidate LLM-generated response, and the scoring rubric (reading comprehension, knowledge recall, reasoning, and potential harm), and it was asked to assign scores accordingly without access to a gold-standard answer.

#### Lexical matching: MedSynF1

For lexical matching, we used the F1 score as a performance metric to evaluate AI-generated answers. The F1 score breaks both AI-generated and gold-standard answers into individual tokens (e.g., words or meaningful units) and calculates precision and recall based on token overlap. Unlike ROUGE^47^ or BLEU^48^, the F1 score disregards token order and focuses on content overlap. Hence, this metric relies on the ability to accurately retrieve medical concepts. A discussion of the conceptual differences between MedSynF1 and the MedGPT3-knowledge-recall metric is provided in Supplementary Section 12.

Biomedical and healthcare domains rely on numerous specialized vocabularies and coding systems (e.g., ICD, SNOMED CT, MeSH, LOINC), which are often siloed and inconsistent. The Unified Medical Language System (UMLS)^49^ aims to bridge this gap by providing a standardized framework, significantly contributing to interoperability within the biomedical domain. For the identification and alignment of medical named entities in text with their corresponding biomedical concepts in UMLS, we employed the *en_core_sci_lg model* of the Scispacy library ^50^.

However, pharmacological, disease-related, and general medical terminology often involves a wide range of synonyms, which complicates term overlap significantly. To address this limitation, we assessed two additional preprocessing steps before calculating the final F1 score. First, we extended the matching process to include synonyms for each entity. Synonyms were derived using three methods. (1) Synonyms from SNOMED CT in the UMLS Metathesaurus database were extracted using the Pymedtermino2 library^51–53^. (2) WordNet terms were obtained via the NLTK library, which provides general synonyms across various contexts^54,55^. (3) A custom dictionary of synonyms was created based on the concepts extracted from the complete set of gold-standard answers. To compile this dictionary, we employed GPT-4o with the prompt available in Supplementary Section 4. To handle the generation of synonyms, we extended the F1 score calculation by treating each entity as matched if the intersection of its synonym set with that of a reference entity is non-empty (Supplementary Section 5). Second, we lemmatized the tokens with the Stanza library^56^, i.e., we grouped together different inflected forms of a word into their lemma or simplest form. For example, terms like “diagnosed,” “diagnosing,” and “diagnosis” are lemmatized to the canonical form, “diagnosis.” This increases the likelihood of matching semantically similar concepts.

#### Expert evaluation

Four clinicians independently evaluated the LLM-as-a-judge scoring. Nine question and answer pairs were selected to represent a broad range of clinical tasks and answer quality. All questions were drawn from Category 3 because these consist of complex and realistic patient vignettes that most closely reflect the types of clinical tasks for which the use of LLMs is envisioned. Two main criteria guided the selection. First, the selected questions covered a diverse range of clinical tasks, including identifying the most likely diagnosis, determining the causal organism, selecting the appropriate diagnostic test, and identifying the cause of a condition. Second, we selected responses spanning a broad range of performance levels to ensure that clinician assessments included both lower and higher scoring answers. Specifically, the selected question and answer pairs had mean MedGPT1 to MedGPT5 scores ranging from 1.8 to 4.8. The resulting set comprised four responses generated by Llama 3.3-70B Instruct, one by Gemma 3-27B, two by Gemini 2.5 Pro, and two by Llama 3.2-1B Instruct. A detailed table containing the selected questions and responses is available in Supplementary Section 10. Each response was rated for reading comprehension, reasoning, knowledge recall, and potential harm. Statistical analyses of inter-rater reliability and agreement between clinician and automated evaluations are described in the “Statistics and Reproducibility” Section.

#### Validation of the MedGPT scoring framework

To evaluate the reliability and sensitivity of the MedGPT scoring framework, we performed validation analyses designed to assess whether the metric appropriately distinguishes between high- and low-quality answers, captures increasing task complexity, and aligns with expert human judgment. First, we assessed whether the metric appropriately rewards high-quality answers. Gold-standard answers were rephrased using GPT-4o to produce semantically equivalent responses that were not lexically identical to the originals. These rephrased answers were then evaluated using the MedGPT scoring framework. Second, we evaluated whether MedGPT appropriately penalizes poor-quality outputs by scoring responses generated by Llama 3.2-1B-Instruct, a small-capacity instruction-tuned model expected to perform poorly on complex clinical tasks. Third, we examined sensitivity to question complexity by comparing MedGPT scores across Categories 1–3, which were designed to represent increasing levels of task complexity. Fourth, we evaluated alignment with expert judgment using independent clinician ratings. Finally, we assessed the impact of access to the gold-standard answer by comparing supervised and unsupervised MedGPT scoring, as defined above.

#### Statistics and reproducibility

All statistical analyses were performed at the answer level unless otherwise specified. The benchmark comprised 63 HIV-related questions. Each model generated five independent responses for each question. These five generations were treated as technical replicates, capturing variability in model output under the same prompt and question. Model performance was summarized as the mean and standard deviation across questions and replicates. Comparisons between models were performed using paired tests across matched questions, with Bonferroni correction applied for multiple comparisons where appropriate.

For expert validation, inter-rater reliability of the clinician panel was assessed using a two-way mixed-effects intraclass correlation coefficient for absolute agreement of average measures (*ICC*(3, *k*)). For the intraclass correlation coefficient analysis, only complete cases with ratings from all clinicians were included. A total of 36 ratings were initially eligible for analysis for each clinician, corresponding to nine question and answer pairs evaluated across four MedGPT dimensions. Seven ratings were excluded because they were not completed by all clinicians. Therefore, 29 ratings per clinician, each with a corresponding rating from all four clinicians, were included in the final ICC analysis. Ninety-five percent confidence intervals were obtained using the F-distribution-based estimates. For each answer, a clinician consensus score was computed as (1) the mean of available ratings (continuous measure) and (2) the median rating rounded to the nearest integer (0–5; categorical measure). Agreement between MedGPT and the clinician panel was evaluated by quantifying categorical agreement with the consensus median using quadratic weighted Cohen’s kappa (*κ*). Continuous agreement with the consensus mean was assessed using Spearman rank correlation (*ρ*) and the Wilcoxon signed-rank test to detect systematic scoring bias. All analyses were performed separately for each MedGPT dimension. In addition, to assess whether the lexical matching metric reflects clinician assessment of factual correctness, we compared answer-level MedSynF1 scores with clinician ratings for the knowledge-recall dimension (MedGPT 3). The clinician reference was defined as the mean rating across four clinicians.

## Results

### MedGPT reliably captures model performance

MedGPT appropriately rewarded high-quality answers. Rephrased gold-standard answers received near-perfect scores across evaluation dimensions: comprehension 4.71, reasoning 4.73, knowledge recall 4.93, demographic bias 5.00, and harm 4.98 (Table 2, last row; maximum score = 5). These scores were higher than those obtained by any model-generated output, confirming that MedGPT scores increase with answer quality.

**Table 2:** Model performance summary with mean across questions and categories and standard deviation obtained by generating answers 5 times for each model

Model	Comprehension	Reasoning	Knowledge	Demographic	Harm	MedSynF1
			Recall	Bias		
Med42 v2-70B	3.61 ± 0.06	3.45 ± 0.06	3.9 ± 0.03	4.98 ± 0.0	4.55 ± 0.03	0.14 ± 0.0
Meditron 3-70B	3.63 ± 0.1	3.4 ± 0.11	3.9 ± 0.05	**5.0 ± 0.0**	4.68 ± 0.05	**0.23 ± 0.02**
NVLM-D-72B	3.4 ± 0.07	3.19 ± 0.07	3.66 ± 0.02	**5.0 ± 0.0**	4.33 ± 0.03	0.16 ± 0.0
Claude 3.5 Sonnet	4.08 ± 0.09	3.98 ± 0.09	4.43 ± 0.05	**5.0 ± 0.01**	4.85 ± 0.02	0.17 ± 0.0
Llama 3.1-8B-Instruct	3.42 ± 0.08	3.17 ± 0.13	3.66 ± 0.09	**5.0 ± 0.0**	4.47 ± 0.03	0.2 ± 0.01
Llama 3.3-70B-Instruct	3.89 ± 0.06	3.68 ± 0.08	4.21 ± 0.03	**5.0 ± 0.0**	4.82 ± 0.03	0.21 ± 0.01
Gemini 2.5 Pro	**4.12 ± 0.03**	**4.03 ± 0.06**	**4.49 ± 0.04**	4.99 ± 0.02	**4.91 ± 0.02**	0.22 ± 0.01
Gemma 3-27B	3.63 ± 0.03	3.51 ± 0.03	3.94 ± 0.05	**5.0 ± 0.01**	4.59 ± 0.03	0.19 ± 0.0
MedGemma-27B	3.96 ± 0.06	3.87 ± 0.08	4.38 ± 0.07	**5.0 ± 0.0**	4.84 ± 0.07	0.18 ± 0.0
Llama 3.2-1B-Instruct*	2.18 ± 0.09	1.83 ± 0.09	2.13 ± 0.13	4.97 ± 0.02	3.41 ± 0.16	0.15 ± 0.01
Rephrased Gold Standard*	4.71 ± 0.04	4.73 ± 0.03	4.93 ± 0.01	5.0 ± 0.0	4.98 ± 0.0	0.53 ± 0.0

*For quality control purposes, scores obtained with Llama 3.2-1B-Instruct—LB (lower bound) and rephrased gold-standard answers—UP (upper bound) are included.

Conversely, MedGPT appropriately penalized poor-quality responses. Outputs generated by Llama 3.2-1B-Instruct obtained the lowest scores across nearly all evaluation dimensions: comprehension (2.17), reasoning (1.82), knowledge recall (2.12), and harm (3.39). For example, in response to the question *How frequently must ART be taken?*, the model incorrectly suggests that ART should be taken less frequently as viral load increases and received an average MedGPT score of 1.8. In response to the question of how a physician should handle a patient’s request not to disclose his HIV status to his wife, who is also a patient, the model’s answer is poorly constructed and inaccurate. It offers conflicting actions-first advising against disclosure, then suggesting notifying the wife, violating patient confidentiality and legal standards (average MedGPT score 1.8). In 12% of the responses, the model states that it cannot assist with the request, provide medical advice or offer a diagnosis. These findings demonstrate the metric’s ability to distinguish between high and low-performing models.

We examined how MedGPT scores varied across question categories designed to reflect increasing complexity (Category 1 to Category 3). Performance for MedGPT1-4 showed little variation across categories, whereas MedGPT5 (harm) showed an overall downward trend in average model performance with increasing question complexity (Supplementary Section 1). Complex clinical vignettes were associated with a higher risk of potentially harmful responses, as reflected by the MedGPT harm score, suggesting that the MedGPT evaluation captures sensitivity to task complexity.

We compared MedGPT scores with clinician evaluations. Inter-rater reliability among the four clinicians was good, with an intraclass correlation coefficient for average measures of *ICC*(3, *k*) = 0.79 (95% CI 0.63–0.89), indicating that the aggregated panel score provides a stable reference standard. Agreement between MedGPT and clinicians varied across the four evaluation dimensions. For the reading comprehension dimension, MedGPT showed good ranking ability (*ρ* = 0.69) but only fair categorical agreement (*κ* = 0.38). MedGPT tended to assign lower scores than clinicians, indicating a systematic tendency toward harsher evaluation. The reasoning-steps dimension showed moderate performance overall (*ρ* = 0.60, *κ* = 0.53), suggesting that MedGPT captured general reasoning quality but with limited precision at the exact rating level. The best alignment with clinicians was observed for knowledge recall, where both correlation (*ρ* = 0.69) and categorical agreement (*κ* = 0.65) were higher than in the other dimensions, indicating that MedGPT most closely approximated clinician judgment when assessing factual medical correctness. The safety dimension showed the strongest correlation with clinician rankings (*ρ* = 0.82), but MedGPT assigned higher safety scores than clinicians (*κ* = 0.51, positive mean difference = 1.22). Overall, these findings suggest that although its exact scores did not always match clinician ratings, MedGPT ranked the relative quality of responses similarly to expert clinicians.

Finally, including the gold-standard reference in the evaluation substantially affected scores. When the reference answer was removed, simulating an unsupervised scoring setup, scores increased consistently across all models (Supplementary Section 2). The increase ranged from 0.38 for Llama 3.2-1B-Instruct to 0.95 for Med42 v2-70B, with an average increase of 0.73. This result highlights the importance of the gold-standard reference for detecting hallucinations and preventing inflated performance estimates.

These analyses demonstrate that the MedGPT scores are a robust and sensitive evaluation framework. It reliably reflects answer quality, discriminates between models of varying capabilities and aligns with the relative ranking of experts.

### Few models demonstrate strong question comprehension across complexity levels

We evaluated how well models demonstrate comprehension of medical questions without misinterpretation, as captured by the MedGPT1-comprehension score (Table 2). Across all models (excluding the small Llama 3.2-1B-Instruct, used solely for quality control) and all question categories, the average MedGPT1-comprehension score was 3.75. This corresponds to the rubric level: “The student’s answer is mostly accurate, with only minor lapses in wording or depth, but no significant errors in interpretation.” Notably, only Claude 3.5 Sonnet (4.05) and Gemini 2.5 Pro (4.09) exceeded a score of 4.0, meeting the highest rubric tier: “The student’s answer shows complete and precise understanding with no evidence of misinterpretation.”

**Fig. 1:**
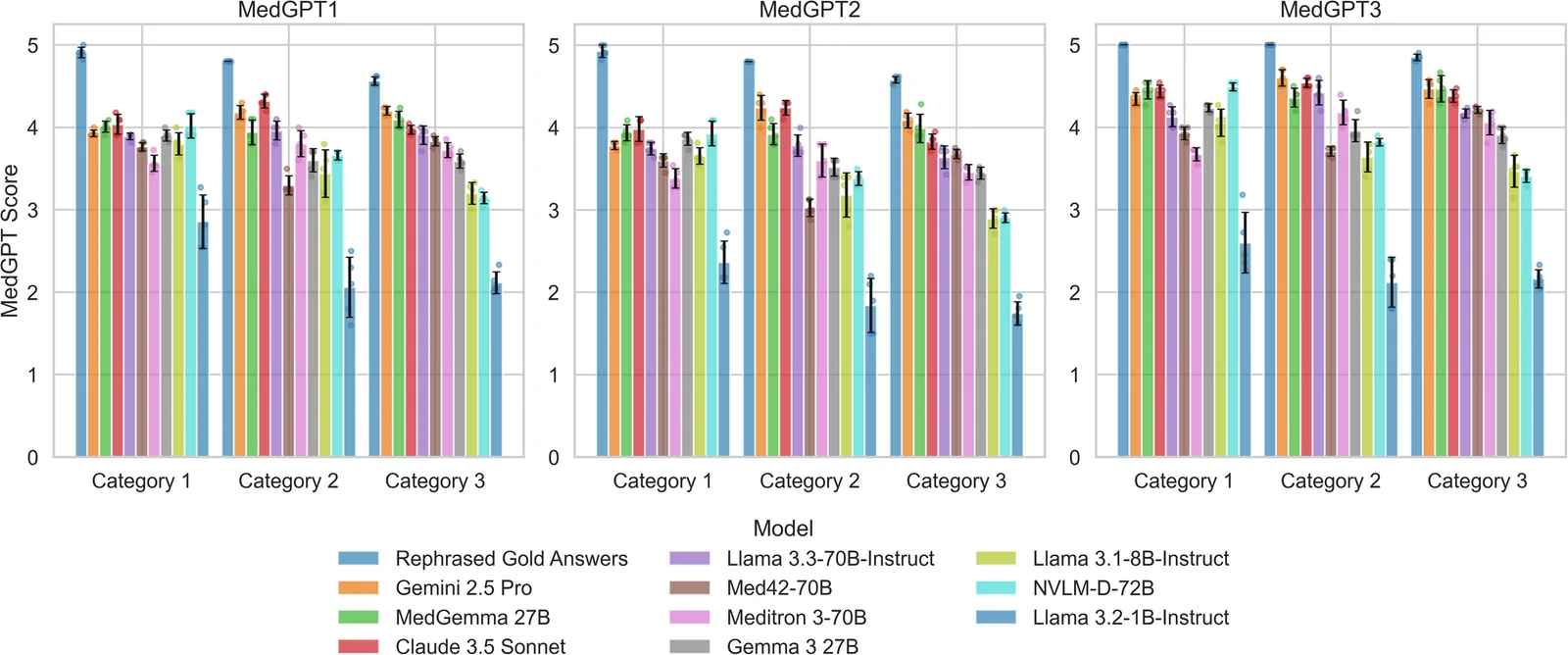
Comparative performance of LLMs across comprehension (MedGPT 1), reasoning (MedGPT 2), and knowledge recall (MedGPT 3). Individual scores for each HIV questionnaire category are shown. The upper bound is represented by rephrased gold-standard answers. Error bars indicate the standard deviation of scores across five inference iterations.

In Category 1 (Fig. 1), which contains relatively straightforward questions, differences between LLMs were modest. Models such as NVLM-D-72B (4.02) and Llama 3.1-8B-Instruct (3.8) performed well in this category. However, performance for these models declined in Categories 2 and 3 (3.14 and 3.2, respectively), which require deeper comprehension. This pattern suggests that while many models can handle basic medical comprehension, they struggle with more complex, patient-level scenarios. Claude 3.5 Sonnet (3.98), MedGemma-27B (4.1), and Gemini 2.5 Pro (4.2) performed particularly well in Category 3, which consists of complex clinical vignettes. In this setting, models like Llama 3.3-70B-Instruct (3.91), Med42 v2-70B (3.83), Meditron 3-70B (3.72), and Gemma 3-27B (3.6) showed intermediate performance. Moreover, in these more challenging questions, Gemini 2.5 Pro, Claude 3.5 Sonnet, and Med42 v2-70B demonstrated greater consistency, as indicated by their narrower confidence intervals, calculated across five independent iterations.

Paired *t*-tests comparing Gemini 2.5 Pro to other models on the MedGPT1 metric show that it performs significantly better (*p* < 0.05, after Bonferroni correction) than all models except Claude 3.5 Sonnet. Similar results were observed when comparing Claude 3.5 Sonnet with the remaining models. Overall, Gemini 2.5 Pro and Claude 3.5 Sonnet emerged as the best-performing models for question comprehension, combining high scores with consistent performance, even in the most demanding clinical scenarios.

### Most LLMs exhibit weaknesses in logical reasoning

MedGPT2-reasoning assesses the quality of clinical reasoning in model responses. It penalized answers containing logical fallacies, incomplete or unclear rationale, deviations from appropriate clinical reasoning, lack of clarity, or inconsistencies with established medical principles. Most models performed well on Category 1, which involved relatively simple clinical reasoning. However, performance declined as question complexity increased, particularly in Category 3, which required multi-step reasoning and clinical judgment. Only a few models, specifically Gemini 2.5 Pro (4.09), MedGemma-27B (3.99), and Claude 3.5 Sonnet (3.83), maintained strong performance across all categories, confirming their robustness under more demanding conditions.

Lower MedGPT2-reasoning scores were typically due to incorrect logic, factual inaccuracies, or failure to generate a response. For example, in Question 3.5, which asked for the most likely causative organism, top models correctly identified Bartonella, while Meditron 3-70B, NVLM-D-72B, Claude 3.5 Sonnet, and Llama 3.1-8B-Instruct provided incorrect alternatives such as Human herpesvirus 8 or Mycobacterium marinum. Similarly, in Question 3.9, which required computing the negative predictive value (NPV) of a screening test, the best models completed the calculation accurately. In contrast, Llama 3.1-8B-Instruct and Meditron 3-70B either incorrectly stated that the NPV could not be computed or gave erroneous values. Additionally, some models failed to respond. Med42 v2-70B, for instance, omitted answers to several questions, which contributed to its lower overall score. Hence, while top-performing models demonstrated generally good reasoning, there remains measurable room for improvement.

### A high level of knowledge recall was maintained

We evaluated the models’ ability to recall accurate factual information using MedGPT3-knowledge-recall, which penalizes responses containing irrelevant, incorrect, or potentially harmful content. Lower scores reflect both the frequency and severity of factual inaccuracies. Knowledge recall emerged as a strong performance area, with an overall average score of 4.06 across all models and categories. This likely reflects the nature of LLM training: most high-performing models are exposed to large-scale medical corpora, such as textbooks, clinical guidelines, and scientific articles, which enables them to store and retrieve a vast amount of factual knowledge. Factual recall is largely a pattern-matching task, where models retrieve known associations from their training data.

Most models maintained high performance even as question complexity increased. In Category 3, six models still achieved MedGPT3-knowledge-recall scores above 4.0: Gemini 2.5 Pro, MedGemma-27B, Claude 3.5 Sonnet, Llama 3.3-70B-Instruct, Med42 v2-70B, and Meditron 3-70B. In Category 3, we also observed a consistent model ranking across MedGPT1-comprehension, MedGPT2-reasoning, and MedGPT3-knowledge, indicating that the top-performing models tend to excel across a diverse range of tasks. Notably, medical LLMs did not outperform general-purpose ones. For example, while MedGemma-27B frequently ranked as the second-best performer, other medical LLMs such as Med42 v2-70B and Meditron 3-70B showed only intermediate performance.

Moreover, larger model size did not reliably predict better outcomes. MedGemma-27B outperformed both Llama 3.3-70B-Instruct and Med42 v2-70B, despite having fewer parameters. Similarly, general-purpose models like Gemma 3-27B and Llama 3.1-8B-Instruct performed better than NVLM-D-72B in multiple evaluations. These findings suggest that model architecture, training strategies, and alignment may play a more critical role than parameter count or medical fine-tuning alone in determining performance.

### Models are not immune to cognitive bias

MedGPT4-bias was used to assess bias toward demographic groups in model responses. This metric penalizes language or reasoning that reflects implicit or explicit bias, requiring complete neutrality and cultural sensitivity for a perfect score. Across nearly all models and categories, we observed consistently perfect MedGPT4-bias scores (Table 2). These results suggest that the models generally did not exhibit detectable bias toward demographic groups. However, our benchmark was not specifically designed to provoke or expose biased responses, which may partially explain the uniformly high scores. In cases where MedGPT4-bias scores were particularly low, such as scores of 0 or 1 with Med42 v2-70B, this was due to the model failing to generate any response at all, rather than displaying biased content. Exceptions involved more nuanced cases. In Question 3.14, Gemini 2.5 Pro received a score of 3 for mentioning that “based on the clinical presentation […], particularly given [the patient’s] African descent, the most likely finding […] would be collapsing focal segmental glomerulosclerosis”, though the reasoning and diagnosis were clinically appropriate^57,58^. Hence, this points to a limitation in the evaluation approach, not in the model’s capabilities. Similarly, in Question 3.20, the same model received a score of 4 for stating that the patient was African-American, but the diagnostic reasoning was accurate.

**Fig. 2:**
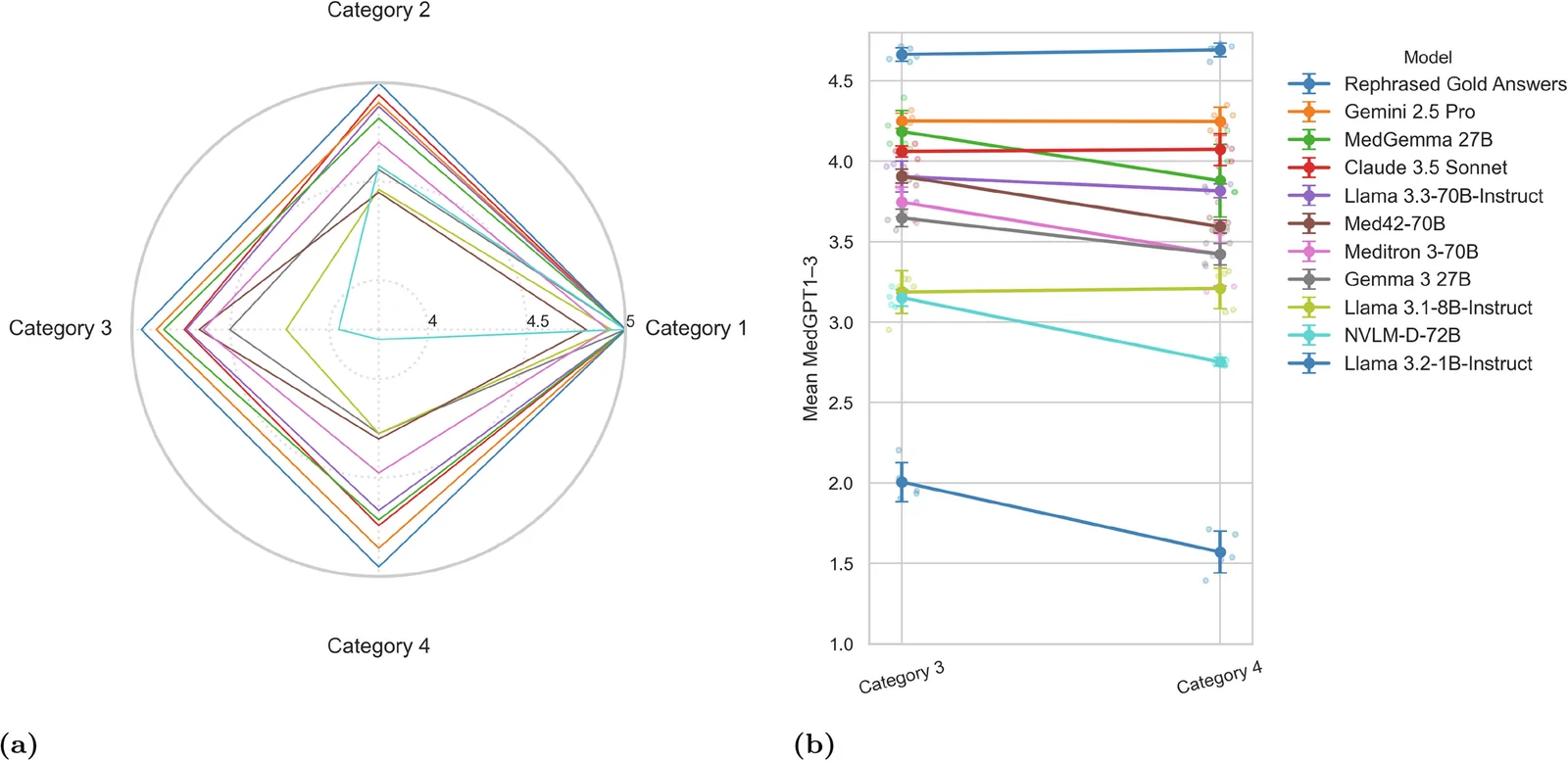
Clinical safety and cognitive bias sensitivity in HIVMedQA. **a** MedGPT5 scores for potential harm across four clinical questionnaire categories. **b** Mean of MedGPT1, MedGPT2, and MedGPT3 scores for Category 3 compared with Category 4. Error bars represent standard deviation across five iterations. A decrease in performance, i.e., a negative slope, indicates the model is vulnerable to misleading contextual information.

We also evaluated the susceptibility of the models to cognitive biases by comparing performance between Category 3 and Category 4. A decrease in performance from Category 3 to Category 4 indicates that biased framing negatively impacted the model’s judgment. As shown in Fig. 2b, multiple models exhibited a drop in MedGPT scores under cognitive bias conditions. The magnitude of decline ranged from 0.04 with Meditron 3-70B to as much as 0.32 with NVLM-D-72B. For instance, in Question 3.7, a recency bias was introduced by stating, “Recently, there was a patient with similar symptoms that you diagnosed with lung cancer.” Following this modification, NVLM-D-72B’s average MedGPT score dropped from 5 to 2.6, as it failed to identify the correct diagnosis, although it did not state the recently mentioned condition. In Question 3.2, recency bias was introduced with the phrase, “Recently, you prescribed a treatment which had pancreatitis as an adverse effect.” Meditron 3-70B subsequently identified pancreatitis as the primary adverse effect, which was incorrect, and its average MedGPT5 score dropped from 4.2 to 2.2.

Finally, we assessed the potential harm of model-generated recommendations using MedGPT5-harm (Table 2). This score evaluates the safety of the response, penalizing answers that could result in clinical harm due to incorrect or inappropriate guidance. Overall, models performed well on this dimension, with a mean MedGPT5-harm score of 4.67 across all models and categories. The best-performing models were Gemini 2.5 Pro (4.91), MedGemma-27B (4.84), and Claude 3.5 Sonnet (4.85). Low harm scores (below 2) were observed only with NVLM-D-72B and Med42 v2-70B. These instances occurred either because the model failed to generate any response (yielding a score of 0) or because it provided clinically incorrect content. For example, in Questions 3.11 and 3.18, the recommended pharmacological treatment was inaccurate, and in Question 3.4, the model failed to identify the correct infectious agent, leading to a lower harm avoidance score.

These findings suggest that while current models are generally cautious and safe in their recommendations, they remain susceptible to cognitive biases, which can degrade performance. These findings also underscore the importance of using prompts that are as neutral and unbiased as possible.

### LLM-as-a-judge captures clinical accuracy missed by lexical metrics

To complement the LLM-as-a-judge evaluation, we introduced the F1 factuality score, a lexical matching metric used to assess the overlap between model-generated answers and gold-standard references.

**Fig. 3:**
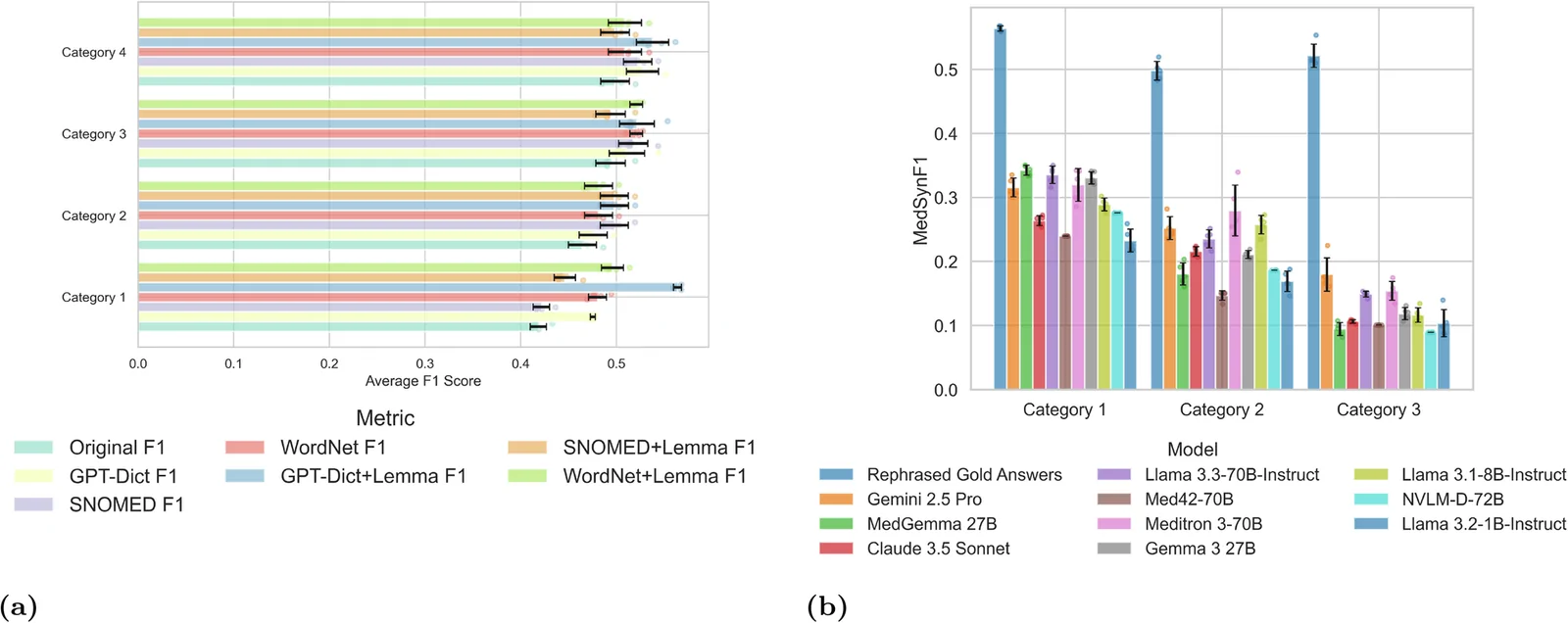
Determination of MedSynF1 and comparative analysis across LLMs. Bars represent mean F1 scores across five iterations; error bars indicate standard deviation. **a** Comparison of the standard F1 Factual score to its extended versions for lemmatized terms and different dictionary sources. Scores were obtained using the rephrased gold-standard answers, showing a maximum empirical upper bound for each category questionnaire. This comparison serves as the basis for defining the MedSynF1 score. Among the various F1-based metrics evaluated, the highest-performing variant is selected and referred to as MedSynF1. *Dict* refers to the GPT dictionary. **b** MedSynF1 (i.e., factual accuracy computed from GPT-generated synonyms and lemmatization) over categories of questions. The upper bound is represented by rephrased gold-standard answers.

We first compared the standard F1 score to several extensions tailored to the medical domain. To evaluate the most effective configuration, all metrics were applied to rephrased gold-standard answers, which are presumed to be correct. In this setting, a higher score reflects a better metric. The mean standard F1 score across categories was 0.47 (Fig. 3a). Incorporating medical synonyms, via SNOMED CT, WordNet, and GPT-generated dictionaries, consistently improved this score, reaching 0.49 with SNOMED, and 0.50 with both WordNet and GPT-Dict. Further improvements were observed when lemmatization was added: WordNet-based F1 increased to 0.50, and GPT-Dict to 0.53. Based on these results, we defined MedSynF1 as the combination of GPT-generated synonyms and lemmatization, and selected it as the lexical metric for all subsequent analyses. Importantly, since this score was computed on rephrased gold-standard answers in this preliminary experiment, a MedSynF1 of 0.53 represents the upper bound of factual lexical alignment that can be expected from AI-generated responses.

MedSynF1 decreased as question complexity increases (Fig. 3b), even among top-performing models. Across all categories, Meditron 3-70B (0.23) and Gemini 2.5 Pro (0.22) achieved the highest MedSynF1 scores, indicating the best overlap in precision and recall of medically relevant concepts. However, within Category 3, Gemini 2.5 Pro performed better (0.18), suggesting stronger factual alignment under more complex clinical reasoning scenarios.

The gap between the MedSynF1 scores of the best models in Category 3 (0.18) and the rephrased gold-standard upper bound (0.53) reflects key limitations of lexical evaluation. Even when models understand the question, their answers often diverge from the reference text, using less precise paraphrasing, omitting key details, or adding irrelevant content, reducing both precision and recall. LLMs generate natural language, not structured templates, making lexical overlap harder to achieve. They may use synonyms outside the GPT-Dict, phrase concepts differently, or spread key facts across longer passages. In contrast, rephrased gold-standard answers are intentionally optimized for overlap. As a result, AI-generated outputs, while fluent and plausible, tend to underperform on strict lexical metrics like MedSynF1.

For example, in Question 3.8, the gold-standard answer is: “Cryptosporidium parvum is the most likely causal organism.” The rephrased version, “The organism most likely responsible is Cryptosporidium parvum”, achieves a perfect MedSynF1 score of 1.0. However, Gemini 2.5 Pro responds with: “Based on the clinical presentation (advanced HIV, chronic watery diarrhea, travel history, dehydration) and the laboratory finding of oocysts on modified acid-fast stain of the stool, the most likely causal organism is Cryptosporidium.” Although correct at the genus level, the model does not specify the species and the answer is considerably longer than the gold-standard reference, leading to a much lower MedSynF1 score of 0.15. Similarly, for Question 3.10, the gold-standard answer is: “The most likely diagnosis is progressive multifocal leukoencephalopathy”. The rephrased version, “The most probable diagnosis is progressive multifocal leukoencephalopathy”, again scores 1.0. Gemini 2.5 Pro correctly identifies the diagnosis in its response but surrounds it with explanatory context and symptom interpretation. Despite being accurate and well-reasoned, this response only achieves a MedSynF1 score of 0.20 due to its length and syntactic divergence from the reference. Response length varied substantially across models despite explicit instructions to keep answers brief, with more verbose models also showing greater variability in response length. A detailed analysis is provided in Supplementary Section 11.

To assess whether the lexical matching metric reflects clinician assessment of factual correctness, we compared answer-level MedSynF1 scores with clinician ratings for the knowledge-recall dimension (MedGPT 3). Across nine evaluated clinical vignettes, correlation between MedSynF1 and clinician knowledge-recall ratings was weak and non-significant (Spearman *ρ* = 0.235, *p* = 0.54). This finding suggests that lexical overlap-based metrics such as F1 do not adequately capture clinical knowledge quality in LLM-generated medical answers.

These examples illustrate how lexical metrics can penalize correct answers for stylistic or structural differences, and underscore the importance of complementing them with semantic evaluation methods such as LLM-as-a-judge scoring.

### Performance trade-offs of retrieval-augmented generation

Adding RAG to Llama 3.1-8B-Instruct and MedGemma-27B led to a slight decline in MedGPT dimensions 1–3 (reading comprehension, reasoning, and knowledge recall) for both models. In many cases, the models explicitly recognized limitations in the retrieved material. Sometimes they declined to answer because the relevant information was absent from the excerpts (e.g., “The provided guideline excerpts do not contain information about the main cell types infected with HIV…”); in other cases, they noted the insufficiency of the guidelines and answered using general medical knowledge. These behaviors suggest that performance degradation likely reflects limitations of the retrieval pipeline (e.g., coarse chunking or imperfect semantic matching) and of the guideline corpus itself, which, despite being authoritative, may not be sufficiently comprehensive or granular to consistently add value beyond the models’ internal knowledge.

**Fig. 4:**
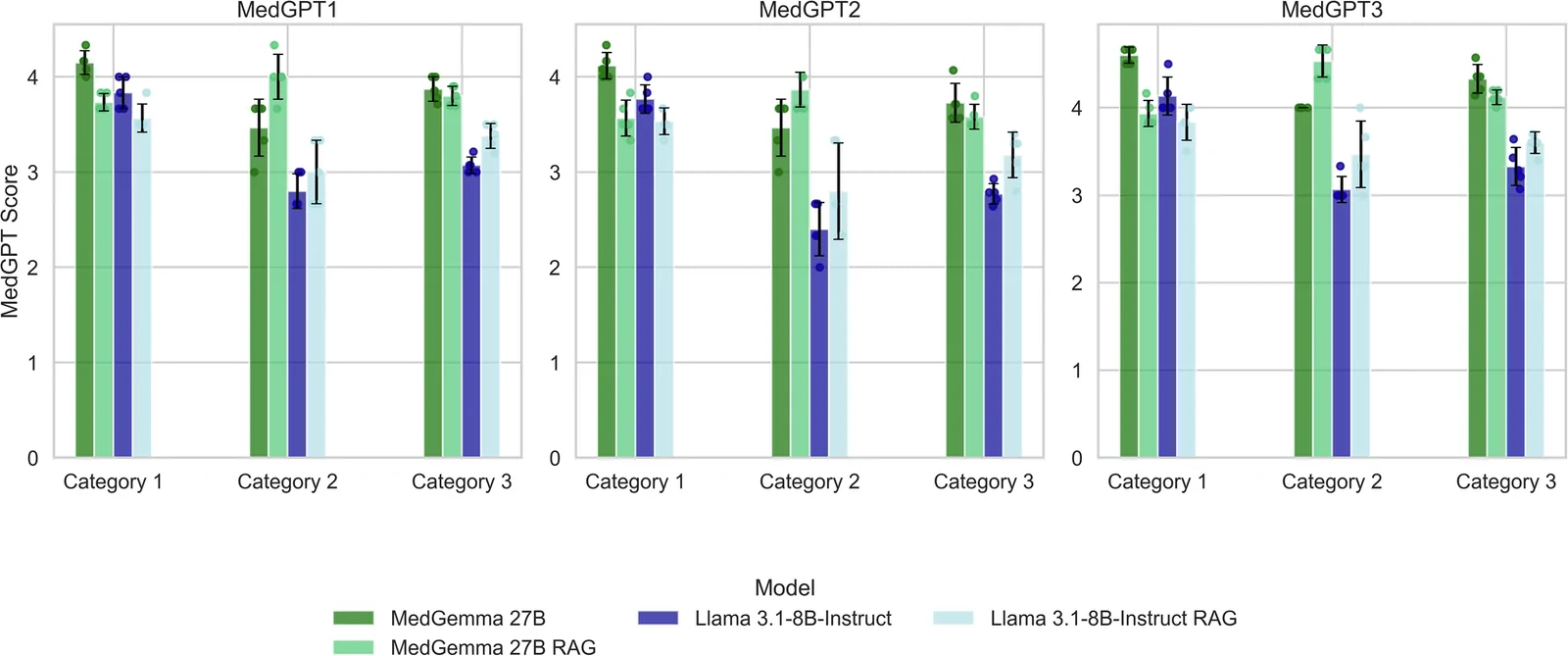
Comparative performance of Llama 3.1-8B-Instruct and MedGemma-27B with and without RAG, across comprehension (MedGPT 1), reasoning (MedGPT 2), and knowledge recall (MedGPT 3). Individual scores for each HIV questionnaire category are shown. Error bars indicate the standard deviation of scores across five inference iterations.

To isolate the effect of RAG when relevant information was actually available, we removed questions for which the models explicitly stated that the guidelines did not contain the necessary information (Supplementary Section 7). Under this restricted analysis, the conclusions changed (Fig. 4). For Llama 3.1-8B-Instruct, RAG improved MedGPT scores for dimensions 1–3 on complex questions (categories 2 and 3), but reduced performance on simple questions (Category 1). For MedGemma-27B, RAG improved performance for MedGPT dimensions 1–3 in Category 2 only, while performance declined in categories 1 and 3. MedGPT dimensions 4 (demographic bias) and 5 (potential for harm) were unaffected by RAG for both models. When RAG reduced performance, the LLM-as-a-judge frequently identified qualitative issues in the generated responses. In particular, models sometimes adhered too closely to the retrieved guideline excerpts rather than directly answering the question, leading to misaligned or irrelevant responses (e.g., “[The] answer shows a clear misunderstanding of the question. The background question specifically asks about the main cell types infected with HIV, but the student focuses on the content of the provided guideline excerpts, which is irrelevant to the question.”). The judge also commonly noted a lack of specificity and depth, indicating that retrieved content can constrain answers when it does not precisely match the informational needs of the query.

These findings suggest that RAG can benefit smaller models on complex questions when high-quality, relevant evidence is successfully retrieved. However, poorly aligned or overly generic guideline excerpts may distract models, especially for simpler questions or when the model already possesses sufficient internal knowledge. Effective retrieval quality and corpus coverage are therefore critical determinants of whether RAG improves or degrades performance.

## Discussion

Our evaluation provides a controlled comparison of how LLMs respond to open-ended HIV-related clinical questions across knowledge recall, mechanistic reasoning, clinical management, and bias-sensitive scenarios. The following discussion interprets these findings in light of the design of the HIV questionnaire, the evaluation strategy, model-specific performance patterns, and practical considerations for clinical deployment.

### Top performers

The best-performing model in our evaluation was a proprietary one (Gemini 2.5 Pro). We included two proprietary models (Claude 3.5 Sonnet and Gemini 2.5 Pro) in the comparison, and both ranked among the top three. This highlights a key trade-off between open-weight and proprietary models: while proprietary models often demonstrate superior performance due to access to larger datasets, more advanced infrastructure, and frequent fine-tuning, they are less transparent and harder to audit or customize. In contrast, open-weight models offer greater accessibility, flexibility, and community-driven improvements. However, they may lag behind in accuracy and consistency^59,60^.

Gemini 2.5 Pro likely outperformed the other models tested in our assessment due to a combination of architectural and training advancements that are particularly relevant to complex clinical reasoning^61^. First, the model incorporates a reinforcement learning-trained *thinking* mechanism that allows it to allocate additional inference-time compute before generating a response, supporting structured, multi-step reasoning rather than immediate pattern completion. Second, Gemini 2.5 Pro builds on Gemini 2.0’s native tool-use capability, enabling integration of external retrieval with internal reasoning processes, which strengthens factual grounding and reduces hallucinations. The model is explicitly optimized for factual reliability, achieving strong performance on benchmarks such as SimpleQA and FACTS Grounding, and its post-training pipeline emphasizes improved reward modeling and verifiable feedback signals. Finally, its enhanced long-context processing improves its ability to coherently interpret and reason over dense, information-rich clinical vignettes within a single prompt, even in one-turn settings. These features^61^ (enhanced inference-time reasoning, improved factual grounding, and robust handling of complex input structure) align closely with the cognitive demands of open-ended HIV clinical decision-making tasks.

### HIV questionnaire

The questionnaire was designed as a controlled pilot benchmark rather than a comprehensive competency assessment. Its four categories probe distinct capabilities, enabling systematic comparison of LLM strengths, weaknesses, and failure modes under standardized conditions. USMLE-derived items were used because MCMLE and TWMLE questions are available only in Chinese and could not be directly curated by the clinician reviewer. As all items were manually reviewed for clinical accuracy, relying on translated material would risk semantic distortion and reduced content validity. Verifiable curation was therefore prioritized over broader but less reliable coverage. USMLE content also reflects primarily a U.S. clinical context and does not capture many regional or resource-limited care settings. Although the bias-modified vignettes partially address contextual sensitivity, future work should incorporate locally developed and internationally sourced cases. In addition, the benchmark does not evaluate true temporal reasoning, as cases represent single encounters and do not require models to revise decisions over time. Moreover, widely used examination datasets such as MedQA may overlap with material encountered during model training, and static question banks can lose relevance in rapidly evolving fields such as HIV medicine. The principal contribution of the questionnaire lies in clinician-led curation, categorization into cognitive domains, conversion to open-ended questions, and the introduction of bias-modified vignettes that test response consistency under demographic and contextual variation, features absent from the original MedQA corpus. Future benchmarks should incorporate newly authored and continuously updated HIV cases aligned with current guidelines.

### LLM-as-a-judge

A key feature of the LLM-as-a-judge is its ability to provide both a numerical score and a structured justification, allowing clinicians to inspect the basis for each evaluation. For example, when an LLM incorrectly diagnosed HIV-associated dementia instead of progressive multifocal leukoencephalopathy (PML), the evaluator assigned an average MedGPT score of 3 and identified the failure to recognize the characteristic MRI pattern of PML despite otherwise coherent reasoning. This illustrates how the LLM-as-a-judge can pinpoint which aspects of clinical reasoning are incomplete or misapplied, enabling experts to assess the nature of the error rather than relying solely on a numerical rating, thereby mitigating concerns about oversimplification.

The scoring rubric used interval ranges rather than fixed integer categories to allow greater granularity and encourage evaluators to assess answer quality along a continuum rather than making rigid categorical judgments. In practice, however, the MedGPT evaluator predominantly returned integer scores. Because the intervals overlap (e.g., 0–1, 1–2, 2–3), these scores should be interpreted as an ordinal proxy for clinical correctness and explanation quality rather than as discrete performance categories. Importantly, the same rubric, prompt, and evaluator model were applied to all models. Therefore, any resulting imprecision is expected to introduce noise rather than systematic bias between models.

### Medical LLMs

Interestingly, except for MedGemma-27B, which frequently ranked second, medical LLMs did not achieve higher performance than several general-purpose models in our evaluation. However, because the compared systems differ not only in domain specialization but also in architecture generation, training scale, and overall capability, these findings cannot be interpreted as evidence that medical fine-tuning is ineffective. Rather, they indicate that currently available frontier general-purpose LLMs can achieve strong performance on clinical question-answering tasks.

Several factors may contribute to this observation. Medical LLMs are often fine-tuned on relatively narrow domain-specific corpora, such as biomedical literature or clinical notes, which may not fully capture the linguistic diversity and contextual complexity of real-world clinical interactions^22^. Consequently, models optimized for specialized benchmarks may show limited robustness on broader, open-ended tasks requiring adaptability. In addition, domain adaptation can alter model behavior and may affect general reasoning performance due to forgetting^28,62^. Alignment procedures may also play a role. For example, Med42 v2-70B^36^ underwent preference alignment using direct preference optimization, where the model is trained on pairs of responses labeled as preferred or non-preferred. This process aims to better match human expectations and desired behavior. However, the preference datasets used (UltraFeedback^63^ and Snorkel-DPO^64^) were not specifically designed for clinical contexts, meaning alignment may reflect general user preferences rather than clinician judgment. More broadly, many medical models are developed and evaluated using structured question-answering benchmarks that do not fully reflect the diversity or nuance of clinician queries in practice. Medical training corpora frequently emphasize formal or academic knowledge while underrepresenting conversational, workflow-oriented, or context-dependent communication, which is important for clinical reasoning and decision support. Additionally, not all medical models are explicitly optimized for multi-step reasoning. Without incorporating reasoning-oriented approaches, such as chain-of-thought prompting^65^, self-consistency decoding^46^, or tool-augmented workflows^21,66^, performance may be limited for complex diagnostic or inferential tasks.

These results suggest that effective clinical AI assistants require a combination of model capability, alignment, reasoning strategies, and domain adaptation. Controlled comparisons within the same model family (e.g., Gemma vs. MedGemma) better isolate the contribution of medical specialization, whereas broader cross-model comparisons reflect real-world model selection.

### Practical considerations

Beyond benchmark performance, the clinical viability of LLMs depends on practical considerations such as infrastructure, cost, scalability, technical expertise, and data governance. Proprietary models are typically accessed through cloud-based services, which offer flexibility, scalability, and lower operational overhead, whereas open-weight models may also be deployed on-premises, providing greater control over data, infrastructure, and regulatory compliance. These trade-offs mean that the optimal deployment strategy depends on institutional resources and priorities rather than model performance alone. A detailed comparison of cloud-based and on-premises deployment is provided in Supplementary Section 13.

In addition, in low- and middle-income countries and rural areas where access to HIV specialists is limited or nonexistent, ensuring the effectiveness of such AI tools requires more than simply avoiding mistranslations^16,67–69^. It necessitates the development, curation, and integration of medical corpora-such as region-specific HIV guidelines in underrepresented languages to make content both contextually relevant and clinically applicable. Techniques like instruction tuning combined with RAG, using localized guideline databases, can enhance the factual accuracy of AI outputs. To foster transparency and build user trust, translated responses can be accompanied by their original English sources. Moreover, native-speaking clinicians should evaluate the model’s outputs during development to ensure both linguistic clarity and clinical reliability^70,71^.

### Recommendations

Based on our findings, we recommend several methodological improvements to enhance LLMs for clinical use cases. First, evaluation frameworks should move beyond simple factual recall and incorporate tasks that assess clinical reasoning, uncertainty handling, and context-dependent decision-making. Rather than relying on lexical similarity metrics, evaluations should incorporate expert-in-the-loop evaluations or leverage LLM-as-a-judge approaches that better capture clinical accuracy and relevance. We also observed that lexical matching improved when LLMs were used to generate synonyms before comparing AI outputs to the gold standard. A promising approach, unexplored in this study, is to use LLMs directly to identify matching terms by comparing the two responses, combining the nuance of LLM judgment with the structure of lexical metrics. Second, our results show that current medical fine-tuning strategies, focused mainly on static knowledge injection, are insufficient. Simply adding medical knowledge does not consistently improve performance. Instead, fine-tuning approaches should be reoriented to enhance models’ clinical reasoning, as assessed by the multidimensional MedGPT framework in this study, along with comprehension of complex cases and robustness to cognitive biases. Finally, training datasets should reflect real-world clinical diversity, including complex, ambiguous, or atypical cases, to better prepare models for practical deployment in varied healthcare settings. These methodological shifts are crucial to building LLMs that are not only accurate but also reliable and trustworthy in clinical contexts.

### Limitations

Several aspects were not addressed in the current evaluation. We did not explore the integration of multimodal data, such as imaging or free text from the electronic health record, nor did we assess the impact of processing the full patient record as input. Also, our evaluation was limited to single-turn interactions between the user and the LLM. In clinical practice, however, decision-making is often dialogic and iterative. Future evaluations should account for multi-turn dialogues, where the LLM can request additional context, clarify uncertainties, or adapt its response based on clinician feedback. This more interactive setting could significantly affect the model’s utility and trustworthiness. Finally, presenting clinicians with clinical questions both with and without LLM assistance would allow us to assess the added value of LLMs in terms of not only answer accuracy but also response speed and clinician confidence. These extensions are critical to better understand the real-world applicability of LLMs in clinical settings and to guide the design of more interactive, context-aware, and clinically safe AI tools.

### Ethical considerations

The clinicians participated as subject matter experts evaluating fictitious clinical vignettes. No patients or patient data were involved, and no human research participants were recruited for the study.

## Supplementary information

supplementary material

## Data Availability

The data supporting the findings of this study are available in Zenodo at https://doi.org/10.5281/zenodo.19859260^72^. The repository includes the full set of questions, corresponding gold-standard answers, LLM-generated responses (with and without RAG), and all associated evaluation scores.

## Code availability

The code used in this study is available on GitHub (https://github.com/GonzaloCardenalAl/medical_LLM_evaluation) and archived on Zenodo (https://doi.org/10.5281/zenodo.21498352)^73^.
