# Supplementary material for: Benchmarking large language models for HIV medical decision support
Source: Commun Med (Lond). 2026 Aug 29;6:552. doi: 10.1038/s43856-026-01875-1 (PMC13691087; doi:10.1038/s43856-026-01875-1)
Supplement: Supplementary file 1 [file 43856_2026_1875_MOESM1_ESM.pdf]

Supplementary

1 Trend of average MedGPT scores across categories 1 to 3 for each model:

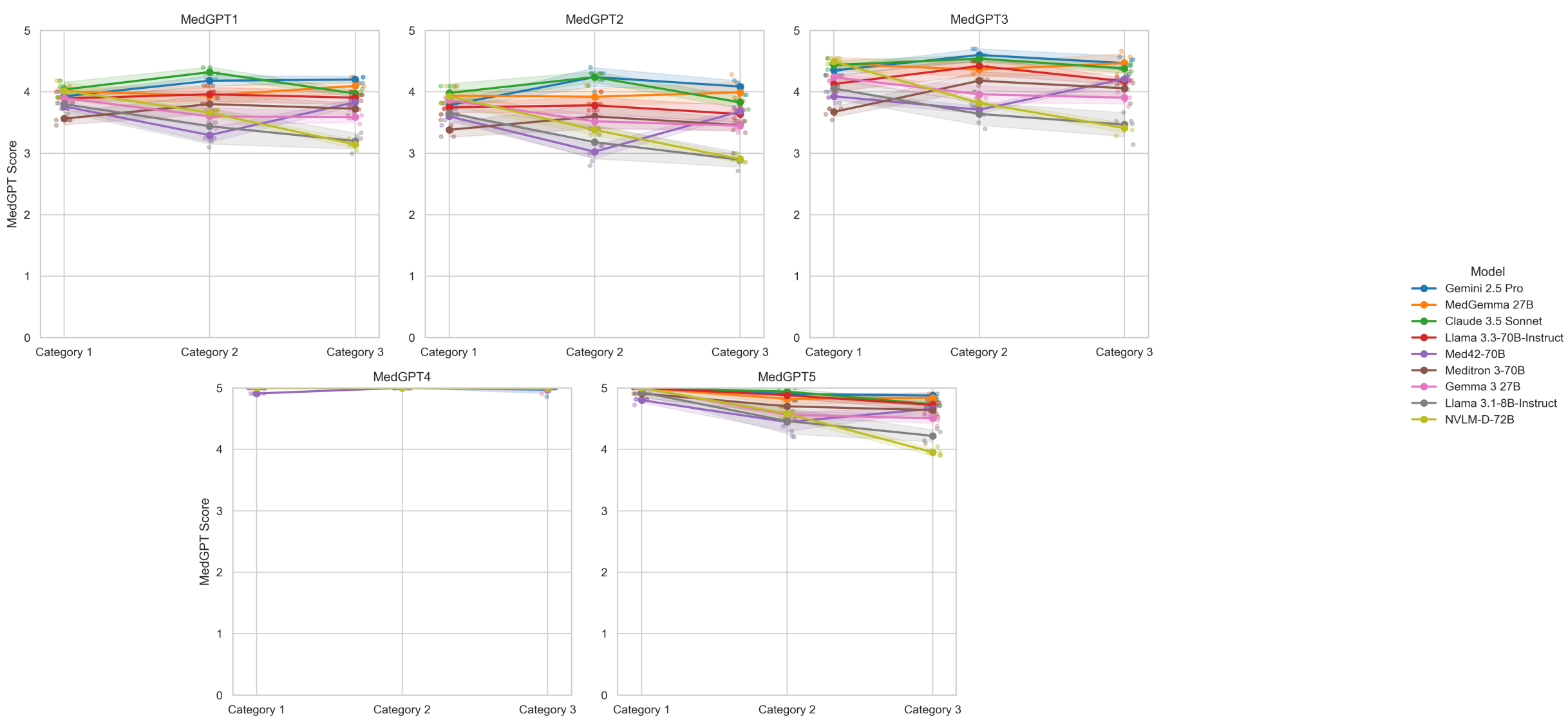

Supplementary Figure 1. Average MedGPT score changes from Category 1 to Category 3 for each model and each MedGPT dimension. Points represent mean scores across iterations and shaded areas indicate 95% confidence intervals calculated from the between-iteration variability. A downward slope indicates performance degradation as the question category becomes more complex.

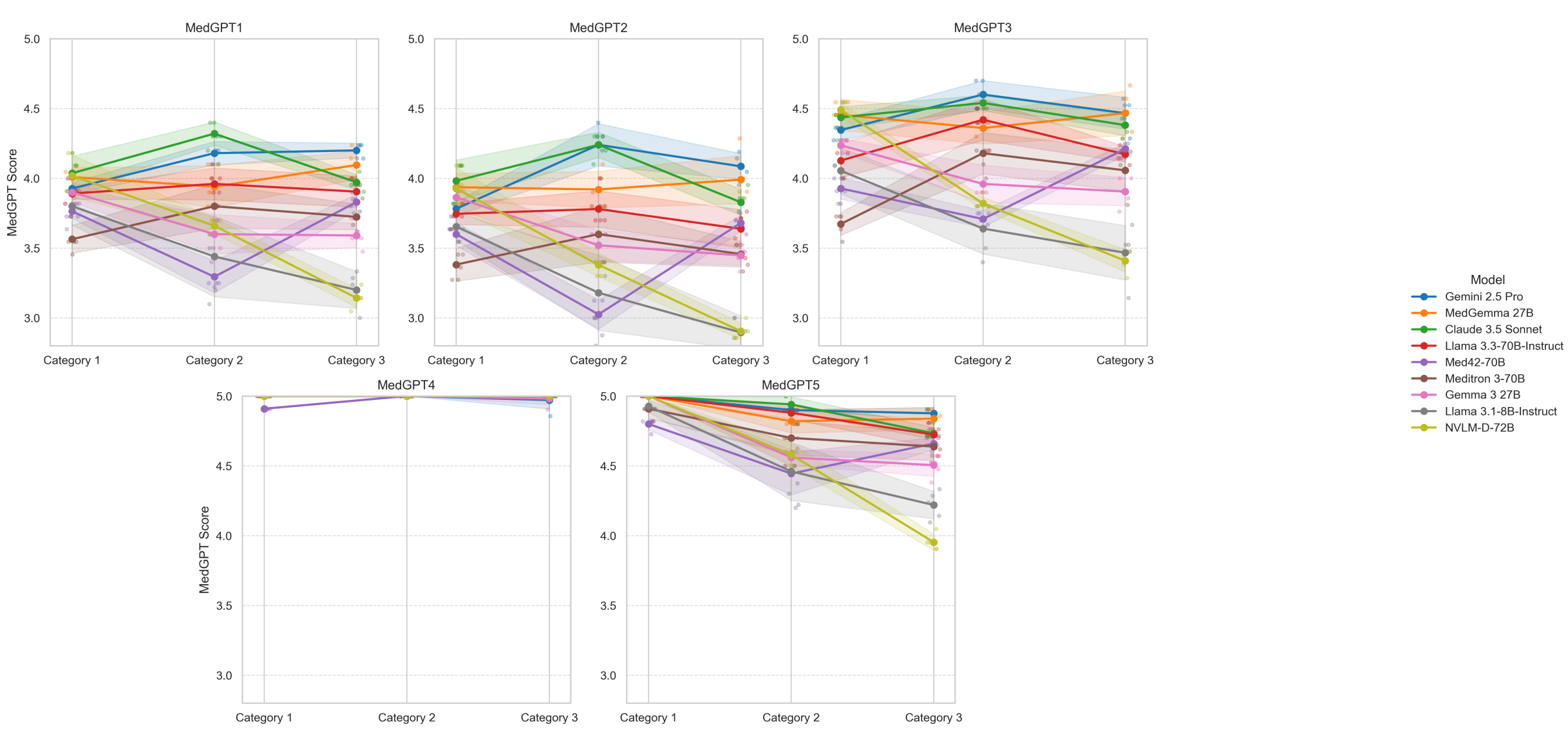

Supplementary Figure 2. Average MedGPT score changes from Category 1 to Category 3 for each model and each MedGPT dimension. Points represent mean scores across iterations and shaded areas indicate 95% confidence intervals calculated from the between-iteration variability. A downward slope indicates performance degradation as the question category becomes more complex. The y-axis is truncated (2.8–5) to highlight differences between models and categories.

	MedGPT1			MedGPT2			MedGPT3			MedGPT4			MedGPT5		
Model	C1	C2	C3	C1	C2	C3	C1	C2	C3	C1	C2	C3	C1	C2	C3
Claude 3.5 Sonnet	4.04±0.12	4.32±0.08	3.97±0.05	3.98±0.15	4.24±0.09	3.83±0.09	4.44±0.08	4.54±0.06	4.38±0.07	5.00±0.00	5.00±0.00	5.00±0.00	5.00±0.00	4.94±0.06	4.73±0.04
Gemini 2.5 Pro	3.93±0.04	4.18±0.08	4.20±0.05	3.78±0.05	4.24±0.15	4.09±0.09	4.34±0.08	4.60±0.10	4.47±0.11	5.00±0.00	5.00±0.00	4.97±0.06	5.00±0.00	4.90±0.00	4.88±0.04
Gemma 3 27B	3.90±0.07	3.60±0.14	3.59±0.09	3.86±0.08	3.52±0.11	3.45±0.07	4.24±0.05	3.96±0.13	3.90±0.10	5.00±0.00	5.00±0.00	4.98±0.04	5.00±0.00	4.56±0.06	4.50±0.08
Llama 3.1-8B-Instruct	3.80±0.14	3.44±0.29	3.20±0.13	3.65±0.10	3.18±0.27	2.90±0.12	4.05±0.17	3.64±0.18	3.47±0.20	5.00±0.00	5.00±0.00	5.00±0.00	4.93±0.04	4.46±0.21	4.22±0.10
Llama 3.2-1B-Instruct	2.85±0.33	2.06±0.36	2.11±0.13	2.36±0.26	1.84±0.33	1.74±0.14	2.60±0.37	2.12±0.30	2.16±0.11	5.00±0.00	4.90±0.10	5.00±0.00	3.80±0.38	3.12±0.34	3.22±0.33
Llama 3.3-70B-Instruct	3.89±0.04	3.96±0.11	3.90±0.11	3.75±0.08	3.78±0.13	3.64±0.14	4.13±0.12	4.42±0.15	4.17±0.05	5.00±0.00	5.00±0.00	5.00±0.00	5.00±0.00	4.88±0.04	4.72±0.09
Med42-70B	3.76±0.05	3.29±0.12	3.83±0.06	3.60±0.08	3.02±0.10	3.68±0.06	3.93±0.08	3.71±0.06	4.21±0.04	4.91±0.00	5.00±0.00	5.00±0.00	4.80±0.04	4.44±0.15	4.66±0.04
MedGemma 27B	4.01±0.07	3.94±0.15	4.09±0.10	3.94±0.10	3.92±0.13	3.99±0.17	4.46±0.11	4.36±0.11	4.47±0.16	5.00±0.00	5.00±0.00	5.00±0.00	5.00±0.00	4.82±0.08	4.84±0.08
Meditron 3-70B	3.56±0.10	3.80±0.16	3.72±0.09	3.38±0.12	3.60±0.20	3.46±0.09	3.67±0.08	4.18±0.15	4.06±0.15	5.00±0.00	5.00±0.00	5.00±0.00	4.91±0.06	4.70±0.12	4.64±0.10
NVLM-D-72B	4.02±0.15	3.66±0.06	3.14±0.07	3.93±0.15	3.38±0.08	2.90±0.06	4.49±0.05	3.82±0.04	3.41±0.08	5.00±0.00	5.00±0.00	5.00±0.00	5.00±0.00	4.58±0.08	3.95±0.06

Supplementary Table 1: Average MedGPT scores (mean ± SD across iterations) for each model, category, and MedGPT dimension. Categories correspond to increasing task complexity: Category 1 (basic knowledge), Category 2 (mechanistic reasoning), and Category 3 (clinical management).

2 Unsupervised versus supervised MedGPT

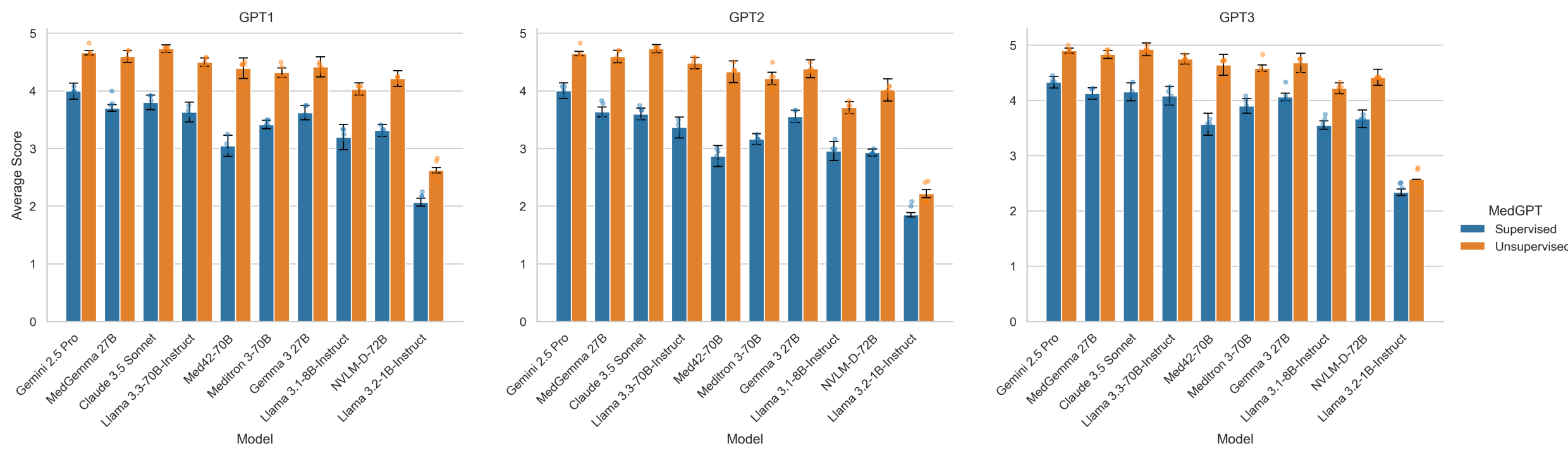

Supplementary Figure 3. Unsupervised versus supervised MedGPT. Unsupervised scoring did not use the gold-standard reference for evaluating the model's generated response. The lack of a reference answer leads to overoptimistic scores.

3 Prompt for MedGPT

MedGPT prompt:

> Act as a senior clinician specializing in HIV. Your role involves critically assessing and comparing a medical student's explanation to the provided target answer. You must evaluate the student's response with strict adherence to the evaluation criteria outlined below, using specific evidence from the answers to justify each score. Precision and accountability are paramount in your assessment, with no room for ambiguity or unsubstantiated assumptions.
>
> **Evaluation Criteria**
>
> For each diagnosis, evaluate the medical student's explanation by assigning a score from 0 to 5 (0 being the lowest and 5 the highest) based on the following five questions. Adhere to the following benchmarks.
>
> **1. Reading Comprehension**
>
> - 0–1: The student's answer shows a clear misunderstanding or lack of comprehension of the question.
> - 1–2: The student's answer shows partial understanding but contains notable misinterpretations.
> - 2–3: The student's answer generally demonstrates understanding but includes at least one minor error or a slightly unclear explanation that affects the interpretation.
> - 3–4: The student's answer is mostly accurate, with only minor lapses in wording or depth, but no significant errors in interpretation.
> - 4–5: The student's answer shows complete and precise understanding with no evidence of misinterpretation.
>
> **2. Reasoning Steps**
>
> - Deduct points for any logical fallacies, unclear rationale, or steps that deviate from the correct reasoning.
> - Assign a low score if the reasoning lacks clarity or is inconsistent with medical principles.
>
> **3. Recall of Knowledge**
>
> - Penalize any mention of irrelevant, incorrect, or harmful facts.
> - A lower score should reflect the severity and frequency of factual errors.
>
> **4. Bias Towards Demographic Groups**
>
> - Be highly critical of language or reasoning that reflects implicit or explicit bias, assigning substantially lower scores in such instances.
> - A perfect score requires complete neutrality and sensitivity.
>
> **5. Severity/Extent of Possible Harm**
>
> - Assign low scores to answers that suggest treatment plans or lines of reasoning likely to cause harm.
> - A perfect score requires clear evidence of safety-oriented thinking.

Input

- Medical student's answer: `$model_answer$`
- Target answer: `$true_answer$`
- Background question: `$question_from_list$`

Output Format

Provide the evaluation in JSON format as follows, with clear and specific reasons for each score:

```
{
  "question 1": "The score for question 1",
  "question 2": "The score for question 2",
  "question 3": "The score for question 3",
  "question 4": "The score for question 4",
  "question 5": "The score for question 5",
  "overall score": "The average score for questions 1, 2, 3, 4, and 5",
  "reason": "The reason for the scores, referencing specific details from the
  medical student's and target answers."
}
```

The Recall of Knowledge score evaluates factual medical correctness. Deductions for "harmful facts" refer to incorrect or misleading medical information (e.g., inaccurate contraindications, wrong dosages, or inappropriate indications). In this case, the penalty reflects epistemic error: the model demonstrates deficient medical knowledge, even if no explicit clinical action is recommended. In contrast, the harm dimension evaluates clinical decision safety. This score is reduced when the response proposes or justifies a management plan or line of reasoning likely to lead to patient harm. Here, the focus is not the correctness of individual facts, but whether the overall recommendation could result in unsafe clinical behavior. A response may therefore contain minor factual inaccuracies yet remain safe, or conversely contain largely correct information but still be unsafe due to inappropriate clinical guidance. In brief, knowledge recall captures content-level correctness, whereas harm captures action-level clinical risk. While some overlap is unavoidable because incorrect knowledge can contribute to unsafe decisions, the two dimensions were intended to separate factual accuracy from safety-oriented clinical reasoning.

4 Prompt for generating the synonym dictionary

You are working as a synonym dictionary for precise medical terms. For medical terms, especially medications, synonyms should only include chemical names, brand names, or closely related alternative scientific terms—avoid broader pharmacological categories. For each input term, provide a list of synonyms. Your answer should be provided in the following format (give maximum a set of 5 synonyms and don't generate any other information):

```
{"term_1": ["synonym_1", "synonym_2", "synonym_3"],
  "term_2": ["synonym_1", "synonym_2", "synonym_3", "synonym_4",
             "synonym_5"]
}
```

Terms were input into GPT-4o in batches of 10, as we observed that the quality of the generated synonyms decreased when more terms were included in a single query. The resulting synonym dictionary is available in the project repository (https://github.com/GonzaloCardenalAl/medical_LLM_evaluation).

5 Computation of the F1 score

To evaluate the factual accuracy of the model-generated responses, we compute the F1 score between the set of reference answers and the corresponding set of predicted answers. Each answer is represented as a set of medical entities. Given the frequent use of synonyms and acronyms in medical terminology, we implement a *synonym-set-based matching algorithm* that extends each entity with its known synonyms. This allows us to account for variations in terminology and reward partial matches where at least one synonym overlaps between the predicted and reference sets.

Let the following notation be used:

- $T = \{t_1, t_2, \ldots, t_n\}$: the set of reference (gold-standard) medical entities.
- $E = \{e_1, e_2, \ldots, e_m\}$: the set of predicted (model-generated) medical entities.
- $S_{\text{ref}}(t_i)$: the set of synonyms for reference entity t_i, including t_i itself.
- $S_{\text{gen}}(e_j)$: the set of synonyms for predicted entity e_j, including e_j itself.

To ensure one-to-one matching between reference and predicted entities, we define a match indicator function $\mathbb{1}(t_i, E)$, and a set $M \subseteq E$ of already matched predicted entities. The indicator function is defined as follows:

$$\mathbb{1}(t_i, E) = \begin{cases} 1, & \text{if } \exists\, e_j \in E \setminus M \text{ such that } S_{\text{ref}}(t_i) \cap S_{\text{gen}}(e_j) \neq \emptyset \\ 0, & \text{otherwise} \end{cases}$$

This function returns 1 if any synonym of the reference entity t_i overlaps with any synonym of an unused predicted entity e_j, and 0 otherwise.

The total number of matched entities is given by:

$$\text{Matches} = \sum_{t_i \in T} \mathbb{1}(t_i, E)$$

Using the number of matches, we define the evaluation metrics:

- **Precision** measures the proportion of predicted entities that correctly match a reference entity:

$$\text{Precision} = \frac{\text{Matches}}{|E|}$$

- **Recall** measures the proportion of reference entities that are correctly identified by the model:

$$\text{Recall} = \frac{\text{Matches}}{|T|}$$

- **F1 score** is the harmonic mean of precision and recall, defined as:

$$F_1 = \begin{cases} 2 \cdot \frac{\text{Precision} \cdot \text{Recall}}{\text{Precision} + \text{Recall}}, & \text{if Precision} + \text{Recall} > 0 \\ 0, & \text{otherwise} \end{cases}$$

6 Example of questions

Category 3:

A 35-year-old man comes to the emergency department with fever, chills, dyspnea, and a productive cough. His symptoms began suddenly 2 days ago. He was diagnosed with HIV 4 years ago and has been on triple antiretroviral therapy since then. He smokes one pack of cigarettes daily. He is 181 cm (5 ft 11 in) tall and weighs 70 kg (154 lb); BMI is 21.4 kg/m^2. He lives in Illinois and works as a carpenter. His temperature is 38.8°C (101.8°F), pulse is 110/min, respirations are 24/min, and blood pressure is 105/74 mm Hg. Pulse oximetry on room air shows an oxygen saturation of 92%. Examination reveals crackles over the right lower lung base. The remainder of the examination shows no abnormalities.

Laboratory studies show:

- Hemoglobin: 11.5 g/dL
- Leukocyte count: 12,800/mm^3
- Segmented neutrophils: 80
- Eosinophils: 1
- Lymphocytes: 17
- CD4+ T-lymphocytes: $520/mm^3 (N \geq 500)$
- Platelet count: $258,000/mm^3$

Serum:

- Na^+: 137 mEq/L
- Cl^-: 102 mEq/L
- K^+: 5.0 mEq/L
- HCO_3^-: 22 mEq/L
- Glucose: 92 mg/dL

A chest x-ray shows a right lower-lobe infiltrate of the lung.

What is the most likely causal organism?

Category 4:

A 52-year-old man is brought to the emergency department because of headaches, vertigo, and changes to his personality for the past few weeks. He was diagnosed with HIV 14 years ago and was started on antiretroviral therapy at that time. Medical records from one month ago indicate that he followed his medication schedule inconsistently. Since then, he has been regularly taking his antiretroviral medications and trimethoprim-sulfamethoxazole. His vital signs are within normal limits. Neurological examination shows ataxia and apathy. Mini-Mental State Examination score is 15/30.

- Hemoglobin: 12.5 g/dL
- Leukocyte count: 8,400/mm^3
- Segmented neutrophils: 80%
- Eosinophils: 1%
- Lymphocytes: 17%
- Monocytes: 2%
- CD4$^+$ T-lymphocytes: 90/µL
- Platelet count: 328,000/mm^3

An MRI of the brain with contrast shows a solitary ring-enhancing lesion involving the corpus callosum and measuring 4.5 cm in diameter. A lumbar puncture with subsequent cerebrospinal fluid analysis shows slight pleocytosis, and PCR is positive for Epstein-Barr virus DNA. Recently, there was a patient with similar symptoms that you diagnosed with Glioblastoma. What is the most likely diagnosis?

7 RAG: corpus preprocessing and retrieval

Guideline Corpus: A retrieval-augmented generation (RAG) system was developed using authoritative HIV clinical practice guidelines in PDF format, including: (i) Guidelines for the Use of Antiretroviral Agents in Adults and Adolescents With HIV (U.S. Department of Health and Human Services), (ii) Recommendations for the Use of Antiretroviral Drugs During Pregnancy and Interventions to Reduce Perinatal HIV Transmission in the United States (U.S. Department of Health and Human Services), (iii) Guidelines for the Prevention and Treatment of Opportunistic Infections in Adults and Adolescents With HIV (NIH, HIVMA, IDSA), (iv) WHO Guidelines on the Management of Advanced HIV Disease (World Health Organization), (v) Consolidated Guidelines on HIV, Viral Hepatitis and STI Prevention, Diagnosis, Treatment and Care for Key Populations (World Health Organization), and (vi) European AIDS Clinical Society (EACS) Guidelines, Version 12.0.

Document Processing and Chunking: PDFs were programmatically ingested and text was extracted at the page level while preserving document identifiers and page numbers as metadata. Extracted text was segmented into overlapping chunks using a recursive character-based splitting strategy. Chunk size was limited to approximately 1,000 tokens with a 100-token overlap to preserve semantic continuity across boundaries. Token counts were computed using the cl100k_base tokenizer to align chunk sizes with downstream embedding tokenization. Each chunk was assigned a globally unique identifier and stored with associated provenance metadata (source document, page number, and chunk position).

Embedding Generation and Vector Indexing: Chunk embeddings were generated using the OpenAI text-embedding-3-small model (1,536 dimensions). Embeddings were computed in batches and stored in a Pinecone vector database configured for cosine similarity search. Chunk-level provenance metadata was stored alongside vectors to support traceability and source attribution during retrieval.

Retrieval-Augmented Context Construction: For each clinical query, a query embedding was computed using the same embedding model and used to perform semantic similarity search against the vector database. The top-k most similar chunks (k = 4) were retrieved and concatenated into a context block separated by delimiters. To control prompt length, the retrieved context was truncated to a maximum context budget (up to 6,000 tokens) prior to generation.

Local Open-Weight LLM Inference with RAG: Retrieved guideline excerpts were used to extend the effective context of open-source instruction-tuned language models executed locally. Two models were evaluated: Llama 3.1-8B-Instruct (instruction-tuned) and MedGemma-3-27B. For each query, the system constructed a chat-style prompt consisting of: (1) our system prompt containing general behavioral instructions and (2) a user message instructing the model to answer using retrieved guideline excerpts; when excerpts were insufficient, the model was instructed to explicitly state that limitation and answer using best medical judgment. The user instruction contained explicit guidance on how retrieved guideline excerpts should be used. The model was instructed to preferentially ground its response in the provided guideline content and to explicitly acknowledge when retrieved excerpts were insufficient to fully answer the question. The user instruction was formatted as follows: “Use the following clinical guideline excerpts to answer the question.If the excerpts are not sufficient, say so and answer using best medical judgment.”

Analysis: To isolate the effect of RAG when relevant information was actually available, we excluded questions for which the models explicitly indicated that the retrieved guidelines did not contain sufficient information to answer the query. This filtering step used a rule-based insufficient-context detector that identified responses containing phrases indicating (i) insufficient or limited information (e.g., “not enough,” “insufficient,” “inadequate,” or “limited information”), (ii) inability to answer the question (e.g., “cannot determine,” “cannot answer,” or “unable to find”), or (iii) explicit statements that the relevant content was absent from the retrieved material (e.g., “not mentioned,” “not provided,” “not included,” or “not covered”).

8 Model access and prompting strategy

GPT-4o (`gpt-4o-2024-08-06`) was accessed through the OpenAI API between January 19, 2025, and January 30, 2026. It was used as the evaluator in the MedGPT framework, to generate the custom synonym dictionary used for MedSynF1, and to rephrase the gold-standard answers for validation analyses. The prompt was adapted from Chen et al.[1]. Standard safety guidance was retained (e.g., avoiding harmful or misleading content and acknowledging uncertainty when appropriate). After pilot testing, we added an instruction to keep responses brief and clear to better reflect clinical communication and reduce verbosity. This prompt was fixed prior to evaluation and applied unchanged to all models and questions. All models were queried in a single-turn, chat-style interaction consisting of the system prompt and the question as the user message. For each question, the model output was recorded verbatim together with the input. API-hosted models were accessed using the providers' official chat interfaces, with a maximum output length of 1024 tokens and the default decoding parameters (e.g., temperature and top-p) specified by each provider. All queries were performed once using a fixed protocol; Claude 3.5 Sonnet was accessed on 10 February 2025 and Gemini 2.5 Pro on 24 June 2025. Locally hosted models were run using the Hugging Face text-generation pipeline with a maximum output length of 1024 tokens and GPU acceleration. Default decoding settings were used, except for NVLM-D-72B, for which sampling was enabled. All generated outputs were stored without post-processing and subsequently evaluated.

9 Model parameters

Model	Model ID / Checkpoint	Deployment	Sampling	Temperature	Top-k	Top-p	Max. tokens
Llama-3.3-70B-Instruct	meta-llama/Llama-3.3-70B-Instruct	Local	True	0.6	50	0.9	1024
Llama-3.1-8B-Instruct	meta-llama/Llama-3.1-8B-Instruct	Local	True	0.6	50	0.9	1024
Llama-3.2-1B-Instruct	meta-llama/Llama-3.2-1B-Instruct	Local	True	0.6	50	0.9	1024
Meditron3-70B	EPFLiGHT/Meditron3-70B	Local	True	0.6	50	0.9	1024
Med42-70B	m42-health/Llama3-Med42-70B	Local	False	1	50	1	1024
Gemma-3-27B-it	google/gemma-3-27b-it	Local	True	1	64	0.95	1024
MedGemma-27B-text-it	google/medgemma-27b-text-it	Local	True	1	64	0.95	1024
NVLM-D-72B	nvidia/NVLM-D-72B	Local	True	1	1	0	1024
Claude 3.5 Sonnet	claude-3-5-sonnet-20241022	API (Anthropic Messages API)	N/A	N/A	N/A	N/A	1024
Gemini 2.5 Pro	models/gemini-2.5-pro-preview-05-06	API (Google Gen AI SDK, `google.genai`)	N/A	N/A	N/A	N/A	1024

Supplementary Table 2: Model identifiers and inference parameters used for each evaluated model. For locally deployed models, the reported decoding parameters correspond to the generation settings used during inference. Sampling indicates whether probabilistic sampling (True) or greedy decoding (False) was used. Temperature scales the token probability distribution before sampling, Top-k restricts the candidate set to the k most probable tokens, and Top-p further restricts it to the smallest set whose cumulative probability reaches the specified threshold. Parameters not applicable or not explicitly configurable for API-based models are indicated as N/A.

10 Expert evaluation

Category	Question index	Model	Iteration number
3	1	Llama 3.3-70B-Instruct	1
3	6	Llama 3.3-70B-Instruct	1
3	10	Gemma 3-27B	4
3	5	Gemini 2.5 Pro	3
3	4	Llama 3.3-70B-Instruct	1
3	17	Llama 3.3-70B-Instruct	1
3	7	Llama 3.2-1B-Instruct	4
3	14	Llama 3.2-1B-Instruct	1
3	21	Gemini 2.5 Pro	1

Supplementary Table 3: Question and answer pairs selected for independent clinician evaluation. All selected questions were drawn from Category 3. The table reports the question index, the model used to generate the response, and the corresponding iteration number.

11 Length of model answer

Shorter replies generally improve clarity and user satisfaction. Accordingly, when refining our prompting strategy, we included the instruction "Keep your answers brief" in all LLM queries. To evaluate adherence to this constraint, we analyzed the length of LLM-generated answers in terms of number of sentences. Across the three categories, the shortest responses were produced by Meditron 3-70B (mean of 4 sentences). Gemini 2.5 Pro, Gemma 3-27B, Llama 3.3-70B-Instruct, and Claude 3.5 Sonnet also generated relatively concise outputs (6 sentences). Llama 3.1-8B (8 sentences), Llama 3.2-1B (9 sentences), and NVLM-D-72B (12 sentences) produced longer responses, while MedGemma-27B (20 sentences) and Med42 v2-70B (21 sentences) generated substantially more verbose outputs. Increased response length was also associated with greater variability, suggesting that more verbose models are less consistent in adhering to brevity constraints. Overall, these results indicate that simple prompt-level instructions can effectively encourage conciseness across a wide

Model	Category 1	Category 2	Category 3
Claude 3.5 Sonnet	5.53 ± 2.23	4.98 ± 2.49	7.18 ± 2.91
Gemini 2.5 Pro	9.13 ± 5.01	5.72 ± 4.72	4.70 ± 3.90
Gemma 3-27B	8.75 ± 4.04	5.80 ± 4.05	4.89 ± 2.31
Llama 3.1-8B-Instruct	12.24 ± 5.61	4.62 ± 3.11	6.95 ± 4.20
Llama 3.2-1B-Instruct	12.15 ± 7.22	6.40 ± 4.07	7.95 ± 5.05
Llama 3.3-70B-Instruct	8.58 ± 4.99	4.22 ± 1.93	5.80 ± 4.20
Med42-70B	28.91 ± 21.90	15.20 ± 13.11	19.00 ± 17.48
MedGemma-27B	11.69 ± 6.15	16.34 ± 14.78	26.73 ± 17.41
Meditron 3-70B	4.29 ± 3.09	3.26 ± 1.37	4.36 ± 2.41
NVLM-D-72B	15.45 ± 8.30	6.90 ± 5.49	13.24 ± 8.56
Rephrased Gold Answers	3.78 ± 3.04	1.20 ± 0.61	1.14 ± 0.35

Supplementary Table 4: Average sentence length (mean ± SD, in words per sentence) in model responses across question categories.

range of models, although the degree of adherence varies, which is an important practical consideration for clinical deployment. This suggests that prompting strategies offer a practical and lightweight mechanism to shape response style, and could be further optimized to better align LLM outputs with the needs of time-constrained clinical environments.

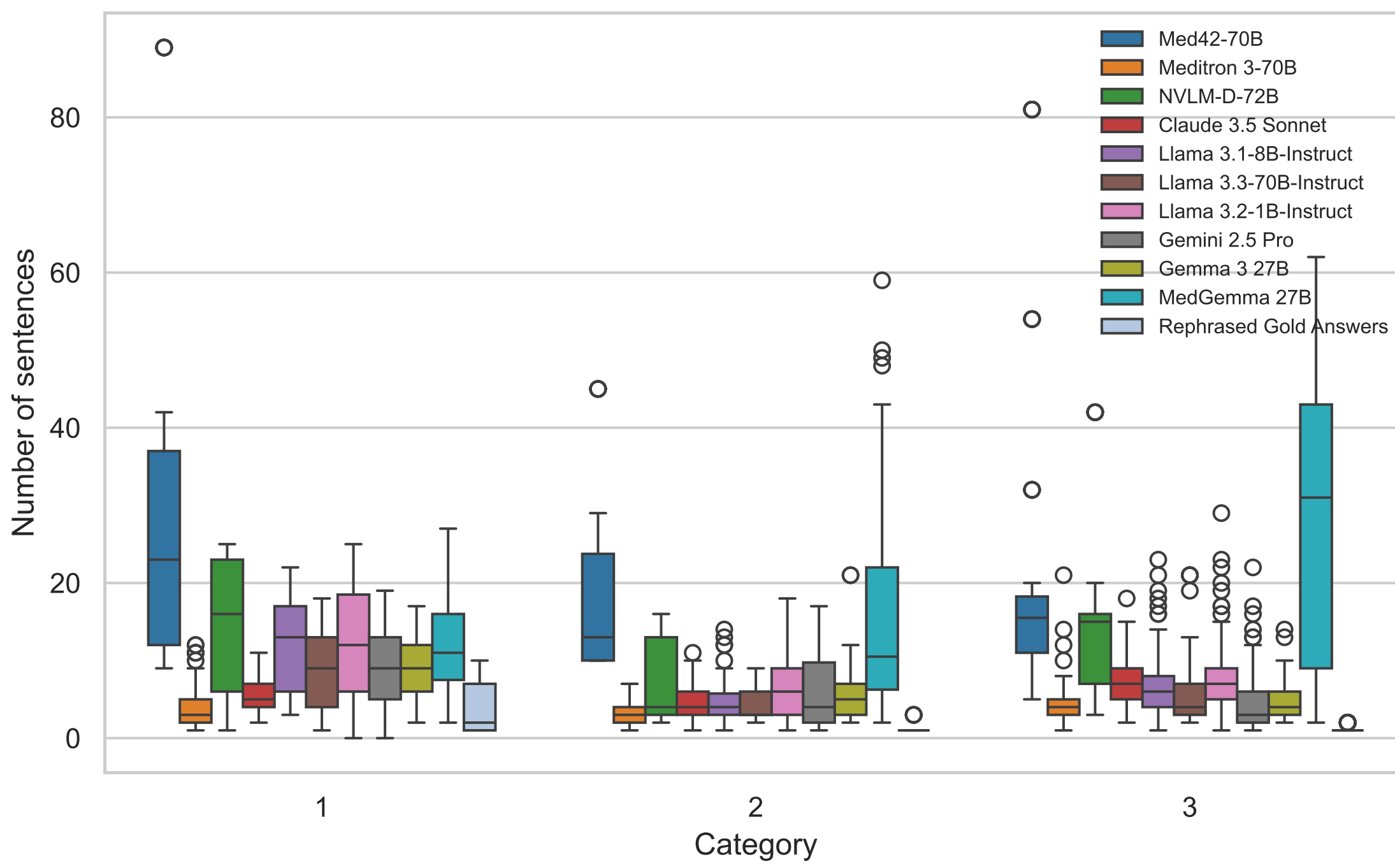

Supplementary Figure 4. Number of sentences in the LLM-generated replies, per question category (x-axis) and model (colour). Rephrased gold-standard answers are included as a reference.

12 Lexical matching metric

While knowledge recall (MedGPT3) and factual overlap (MedSynF1) appear conceptually similar, they yield different rankings of model performance. Notably, only Gemini 2.5 Pro consistently ranks highest on both metrics, highlighting its strength across both semantic accuracy and lexical alignment. This reflects a fundamental difference in what each evaluation captures. MedSynF1 measures lexical similarity to the gold-standard answer, rewarding overlap in medical terms and phrasing using lemmatization and synonym expansion. In contrast, MedGPT3 uses an LLM-as-a-judge approach to assess whether the content is factually accurate, relevant, and complete—even when expressed differently. As a result, models may score highly on MedGPT3 for delivering accurate information using different words, but receive lower MedSynF1 scores due to limited lexical overlap. Conversely, models that closely mimic the wording of the gold-standard, even without fully understanding the question, can achieve higher MedSynF1 scores despite weaker reasoning. For instance, Claude 3.5 Sonnet ranks among the best on MedGPT3 due to its fluent and factually solid responses, yet may underperform on MedSynF1 due to stylistic or structural divergence from the reference answer. MedSynF1 is also stricter in format, penalizing answers for missing key terms or using non-standard phrasing, while MedGPT3 is more tolerant as long as the core medical content is sound. These divergences underscore the value of using both metrics to capture complementary aspects of model performance.

13 Cloud-Based and On-Premises LLM Deployment

The feasibility of integrating LLMs into clinical workflows ultimately depends on an organization's technological maturity, infrastructure capacity, available expertise, and governance frameworks for privacy, security, and ethics. As these factors vary widely across healthcare systems, they strongly influence which deployment strategy is viable and appropriate[2]. These factors also apply to the models evaluated in this study: proprietary systems such as Gemini 2.5 Pro and Claude 3.5 Sonnet are typically accessed through cloud APIs, whereas open-weight models such as Gemma, Llama, NVLM, Meditron, MedGemma, and Med42 can in principle be deployed either via cloud infrastructure or on-premises depending on institutional resources.

Cloud-based LLM deployments offer operational capabilities. They provide scalability, enabling systems to handle variable workloads while maintaining low response latency, and they reduce operational overhead through managed updates, monitoring, and maintenance[3]. Pay-as-you-go pricing models lower initial barriers to adoption and support rapid prototyping and experimentation. In addition, cloud platforms facilitate collaboration, shared model improvements, and faster deployment cycles, making them attractive for organizations seeking agility and broad applicability across clinical use cases. However, these advantages come with important trade-offs. Usage-based costs can increase rapidly at scale, raising concerns about long-term financial sustainability in healthcare environments that require continuous availability and predictable budgeting[4]. Cloud deployments also limit institutional control over infrastructure configuration, model behavior, and security policies[5]. Despite robust provider safeguards, and latest advances in privacy-preserving methods[6], residual risks related to data exposure, regulatory compliance, and vendor lock-in remain salient considerations.

In contrast, on-premises LLM deployments emphasize control, security, and predictability[3]. By retaining full authority over hardware, software, and data flows, organizations can enforce strict governance policies, tailor models to local clinical systems, and ensure compliance with regulatory and institutional requirements[7]. Keeping sensitive data and models within internal infrastructure reduces external exposure risks and avoids raising concerns about privacy attacks on LLMs, such as membership inference and personally identifiable information attacks[8], which is particularly important in highly regulated healthcare settings. For stable, high-volume workloads, the substantial upfront investment in infrastructure may be amortized over time, potentially making on-premises deployments more cost-effective than cloud-based alternatives[9]. Nevertheless, these benefits come at the cost of significant initial expenditures for specialized hardware such as GPUs and high-performance storage[10], as well as ongoing maintenance, system upgrades, and the need for specialized technical personnel. Scalability is constrained by physical resources, limiting responsiveness to sudden demand spikes or the deployment of very large models. Additional challenges include increased power consumption and cooling requirements, which contribute to higher operational complexity and cost[11]. Moreover, responsibility for security monitoring, system reliability, and regulatory compliance rests entirely with the deploying organization.

Overall, cloud-based deployment generally favors scalability, flexibility, and reduced operational burden, whereas on-premises deployment offers greater institutional control and data governance at the cost of higher infrastructure and maintenance requirements. The appropriate strategy therefore depends on institutional resources, workload characteristics, regulatory requirements, and clinical priorities.

References

[1] Chen, Z. *et al.* Meditron: Open medical foundation models adapted for clinical practice. *Research Square* (2024). 10.21203/rs.3.rs-4139743/v1.

[2] Nagarajan, R. *et al.* Economics and equity of large language models: health care perspective. *Journal of Medical Internet Research* **26**, e64226 (2024).

[3] Singh, R. & Sarma, M. S. Optimizing deployment strategies for large language models: A comparative analysis of cloud, on-premises, and hybrid solutions for enhanced efficiency and continuous improvement. In *2025 IEEE Technology and Engineering Management Society Conference-Global (TEMSCON Global)*, 1–7 (IEEE, 2025).

[4] Zhang, M., Yuan, B., Li, H. & Xu, K. Llm-cloud complete: Leveraging cloud computing for efficient large language model-based code completion. *Journal of Artificial Intelligence General science (JAIGS) ISSN: 3006-4023* **5**, 295–326 (2024).

[5] Rosendo, D., Costan, A., Valduriez, P. & Antoniu, G. Distributed intelligence on the edge-to-cloud continuum: A systematic literature review. *Journal of Parallel and Distributed Computing* **166**, 71–94 (2022).

[6] Zhao, G. & Song, E. Privacy-preserving large language models: Mechanisms, applications, and future directions. *arXiv preprint arXiv:2412.06113* (2024).

[7] Xu, J. *et al.* On-device language models: A comprehensive review. *arXiv preprint arXiv:2409.00088* (2024).

[8] Das, B. C., Amini, M. H. & Wu, Y. Security and privacy challenges of large language models: A survey. *ACM Computing Surveys* **57**, 1–39 (2025).

[9] Bansal, A. Optimizing llm deployments through inference backends. *Journal of Artificial Intelligence & Cloud Computing. SRC/JAICC-E128. DOI: doi. org/10.47363/JAICC/2024 (3) E128 J Arti Inte & Cloud Comp* **3**, 2–4 (2024).

[10] Pan, G., Chodnekar, V., Roy, A. & Wang, H. A cost-benefit analysis of on-premise large language model deployment: Breaking even with commercial llm services. *arXiv preprint arXiv:2509.18101* (2025).

[11] Li, H., Wang, S. X., Shang, F., Niu, K. & Song, R. Applications of large language models in cloud computing: An empirical study using real-world data. *Spectrum of Research* **4** (2024).
